# Rapid and energy-efficient ultra-large library screening for drug discovery on a SpiNNaker2 neuromorphic chip

**DOI:** 10.1038/s42004-026-02122-3

**Published:** 2026-07-21

**Authors:** Johnny Alexander Jimenez Siegert, Florian Kelber, Bernhard Vogginger, Paul Eisenhuth, Max Beining, Vivian Ehrlich, Johannes Partzsch, Christian Mayr, Jens Meiler

**Affiliations:** 1 Institute for Drug Discovery, Leipzig University, Leipzig, Germany; 2 Center for Scalable Data Analytics and Artificial Intelligence (ScaDS.AI), Dresden/Leipzig, Germany; 3 School of Embedded Composite Artificial Intelligence (SECAI), Cooperation of University Leipzig and TU Dresden, Dresden/Leipzig, Germany; 4 Institute of Circuits and Systems, Dresden University of Technology, Dresden, Germany; 5 Center for Structural Biology, Vanderbilt University, Nashville, TN USA; 6 Department of Chemistry, Department of Pharmacology and Institute of Chemical Biology, Vanderbilt University, Nashville, TN USA; 7 Faculty of Mathematics and Informatics, Faculty of Chemistry, Leipzig University, Leipzig, Germany

**Keywords:** Virtual screening, Cheminformatics, Cheminformatics

## Abstract

The virtual screening of make-on-demand small molecule libraries can prioritize drug candidates for rapid experimental validation to accelerate pre-clinical drug discovery. As ultra-large libraries grow to billions of compounds, exhaustive screening incurs prohibitive costs and energy consumption. We address this challenge with the neuromorphic SpiNNaker2 system, designed for massively parallel AI tasks. Here, we show the implementation of a ligand-based screening pipeline on a 152-core SpiNNaker2 chip. We adapted feed-forward neural networks trained on 2D molecular descriptors to screen 19 billion molecules from the Enamine REAL space. We benchmarked our approach against an NVIDIA Jetson Orin Nano, a GPU-accelerated low-power AI system. Inference on the SpiNNaker2 chip was approximately 4 times faster, yielding 60% higher overall throughput. Meanwhile, SpiNNaker2 consumed about 86% less energy. These results provide a foundation for the deployment of SpiNNaker2 high performance computing clusters and establish application-specific hardware as a scalable and sustainable avenue for cheminformatics.

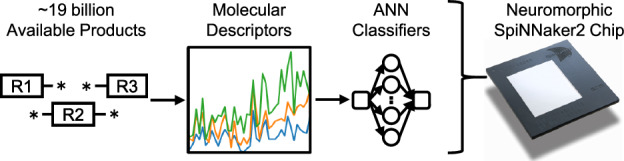

## Abstract (web summary)

The challenge of efficiently exploring ultra-large chemical libraries for drug discovery is exacerbated by the prohibitive costs and energy demands of exhaustive screening. Here, the authors demonstrate the implementation of a ligand-based screening pipeline on a 152-core SpiNNaker2 chip, achieving 60% higher throughput and 86% lower energy consumption than an NVIDIA Jetson Orin Nano system.

## Introduction

Modern drug discovery is a complex and time consuming process, often taking over a decade from start to marketable drug. A high potential for time and cost savings lie in the preclinical stage^1^, where hit molecules binding to pathologically relevant proteins are identified and optimized for potency, selectivity, toxicity and drug metabolism and pharmacokinetic (DMPK) properties, before entering clinical trials^2^. Next to endogenous ligands, natural products or previously identified chemical probes, initial hit molecules are often identified by high throughput screening (HTS) of thousands of small molecules for activity in biophysical assays^3,4^. HTS campaigns are costly and limited to the tiny chemical space physically available at the screening center (typically 10^5^ − 10^6^ molecules) when compared to the drug-like chemical space that is estimated to be around 10^60^ ^5^. With the emergence of (1) artificial intelligence (AI)/deep learning (DL) computational algorithms, (2) ultra-large make-on-demand compound libraries of more than 10^10^ molecules and (3) improved hardware architectures, we experience a renaissance of virtual high-throughput screening as is dubbed Ultra-Large Library Screening (ULLS) to identify promising compounds in silico for experimental validation.

Computer-aided drug discovery methods (CADD) can be grouped into structure-based (SB) methods, which incorporate 3D protein structures (for example through docking), and ligand-based (LB) methods, which learn to infer features from sets of small molecules with determined properties like activity (or lack of activity) towards a specific protein target^6^. SB-CADD methods usually have a higher computational cost, while LB-CADD methods are easier to scale up to billions of molecules. Both approaches have been transformed by advances in machine learning (ML). ML-based quantitative structure activity models (QSAR) have long become one of the most common LB-CADD methods^7,8^. Due to their reliance on target-specific binding data, LB-CADD methods are usually restricted to targets with large amounts of experimental data. QSAR models using artificial neural networks (ANNs) have recently been applied as active learning components to accelerate structure-based screening^9–11^. SB-CADD is benefiting from the success of AI/DL methods such as AlphaFold^12^ and RoseTTAFold^13^ in predicting protein structures for most target proteins, in case no experimentally determined structure is available for the conformation of interest^14,15^. More recently, DL-based docking^16^ and co-folding^17–19^ methods have been suggested to predict receptor-ligand binding structures and affinities^20^. While generalizability and physical plausibility remain challenges for current generation DL methods^21,22^, their inherent treatment of protein flexibility and independence from traditional scoring functions make them a potentially useful tool in ULLS alongside molecular docking^23,24^.

The rapid expansion of make-on-demand small molecule libraries for ULLS is driven by academic research into combinatorial chemistry and companies perfecting the technologies^25–27^. The combinatorial chemical spaces readily accessible have grown to more than 10^10^ by increasing lists of building blocks or “synthons” and associated chemical reactions with synthesis success rates of 80–90%^28^. Larger libraries for ULLS increase the chance of finding higher-affinity hit compounds with possibly more diverse chemical scaffolds as long as the molecules can be ranked correctly by the scoring function^29,30^. The ultra-large library employed herein, the Enamine REAL space, currently encompasses more than 60 billion make-on-demand molecules^27,31^. Common approaches to navigate such chemical spaces more efficiently apply ligand-based heuristics^32^, such as QSAR models^9,11^ or evolutionary algorithms^33,34^, to iteratively dock a subset of molecules and extrapolate to the rest of the library, instead of docking the entire library. These methods run the risk of missing the best scoring scaffolds, however they have been applied successfully to identify small molecule ligands. While billion-scale docking campaigns are becoming more common, they remain computationally challenging, requiring millions of CPU hours^30,35,36^. As ultra-large libraries are expected to continue their rapid expansion, brute-force docking will become harder to scale up.

The development and adoption of CADD methods has been enabled by the widespread availability of high-performance computing (HPC) resources, including graphics processing units (GPUs)^1^. Next to the acceleration of traditional methods such as molecular dynamics simulations^37^ and molecular docking^38^, GPUs have enabled the myriad of DL-based methods transforming LB- and SB-CADD^39^. ULLS campaigns can be scaled up with large heterogeneous HPC clusters, if access to enough GPUs and the associated energy consumption are given. Less commonly, CADD tasks have been accelerated with field-programmable gate arrays (FPGAs). Examples include molecular docking with Vina-FPGA^40,41^, calculations in Molecular Dynamics (MD) simulations^42^ and similarity search^43^. While FPGAs improved on CPUs and GPUs in some tasks, i.e., Vina-FPGA consuming less than half the energy of a GPU implementation, they are less widely adopted and require more effort to optimize for^44^. As the size of chemical libraries increases, the continued success of ULLS campaigns depends on HPC capabilities, including the development of new hardware architectures to not only provide the computational power, but do so in an energy-efficient way^45–47^.

One step further, with the potential to maximize energy efficiency and ensure high throughput, is to employ application-specific integrated circuit (ASIC) hardware. As an example for simulation of molecular dynamics, the Anton2 architecture proves said advantages^48^. For ANNs and event-based applications, the SpiNNaker2 neuromorphic system introduces many interconnected and efficient small nodes to tackle challenging tasks through highly-parallel scalability^49,50^. One SpiNNaker2 system-on-chip provides 152 processing elements (PEs), each comprising a general-purpose ARM Cortex M4F core equipped with dedicated accelerators, including a machine learning accelerator (MLA)^51,52^. Instead of employing an integrated central operating system, the PEs can be programmed with embedded code from a host PC over Ethernet and run independently in parallel of each other. For larger executions, SpiNNaker2 chips have off-chip DRAM access and can be interconnected through specialized chip-to-chip (c2c) links. The system has proven to run multiple applications with gains in efficiency, including, amongst others, traditional and bio-inspired AI^53–57^, optimization algorithms^58^ and quantum simulation^59^. As of now, the efficiency of ULLS was not explored on ASIC hardware, including SpiNNaker2 specifically.

**Fig. 1:**
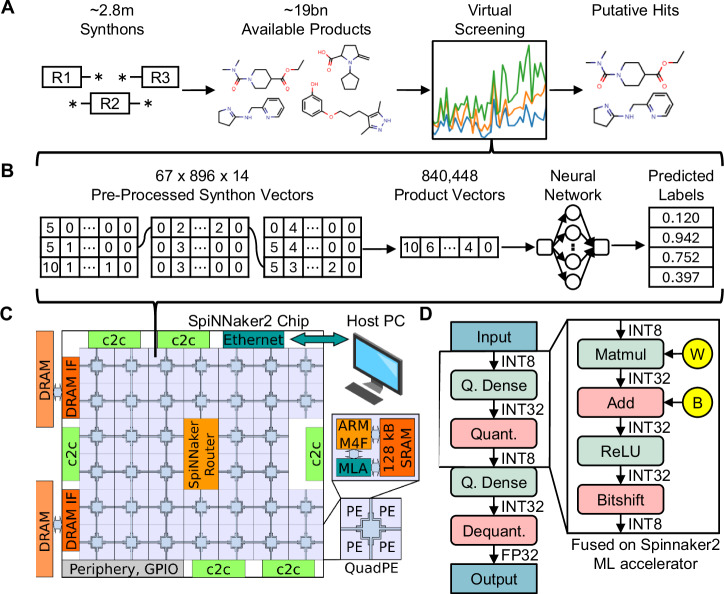
Ultra-large library screening on a SpiNNaker2 chip. **A** The Enamine REAL space makes billions of molecules available for virtual screening and rapid experimental validation of putative hits. **B** We have implemented a ligand-based screening pipeline with a combinatorial descriptor calculation and a quantized ANN inference for a library of 19 billion molecules from the Enamine REAL space. **C** We benchmarked this pipeline on a SpiNNaker2 chip with 152 processing elements. Each processing element independently fetched inputs, ran descriptor calculation and inference for a set of products, until all combinations were evaluated. **D** For inference on the SpiNNaker2 chip, we used a neural network with 32 hidden neurons. We quantized the models from float32 to int8 precision to use int8 matrix multiplication on the SpiNNaker2 machine learning accelerators. By modifying the following activation (ReLU) and int32-to-int8 quantization (fixed-width bitshift), the entire inference could be off-loaded to the MLA.

Here, we present the implementation of an LB-ULLS approach with feed-forward ANNs on the massively parallel SpiNNaker2 architecture, shown in Fig. 1. To reduce off-chip DRAM access, we introduced a scheme to calculate 2D molecular descriptors for combinatorial libraries. We quantized and optimized our neural networks for execution on SpiNNaker2 while maintaining predictive performance. We benchmarked our approach using a handwritten C implementation on a 152-core SpiNNaker2 chip to screen a library of 19 billion molecules from the Enamine REAL space. As a comparison, we implemented the same benchmark on an Nvidia Jetson Orin Nano, a low-power embedded system with a Tegra SoC incorporating a 6-core ARM CPU, an Ampere GPU, and 8 GB of shared system RAM. We found that inference on the machine learning accelerators of the SpiNNaker2 chip was more than four times as fast as on the Ampere GPU. Including input preparation we achieved 60% higher throughput on SpiNNaker2 while consuming about 86% less energy.

Overall, our results demonstrate that SpiNNaker2 offers scalable performance comparable to current GPU-accelerated systems with considerably lower energy consumption. Based on our implementation, we highlight optimizations and hardware features, including the scalable high-throughput data transfer mesh, which will be crucial for future applications on the SpiNNaker2 architecture.

## Results

### Neural network training and quantization

As a realistic ligand-based drug discovery workload, we used ANN classifiers trained to predict biological activity from molecular descriptors. We trained our models on nine datasets^60,61^ of small molecules labeled as active or inactive against a given protein target based on curated PubChem HTS assays, see Table 1. For each dataset, we used the Biochemical Library (BCL) to calculate 2D molecular descriptors (BCL::Mol2D)^62^. We trained ANNs with one hidden layer of 32 neurons, largely following the training procedure and model architecture previously used to establish a baseline performance of BCL::Mol2D descriptors on the same datasets. In order to reduce memory consumption and take advantage of accelerated int8 matrix multiplication on the SpiNNaker2 MLA, we quantized the trained models from float32 to int8 precision via quantization-aware training^63^. During quantization, an int32-to-int8 quantization step was introduced to transform the int32 output of the hidden layer into the int8 input of the output layer. This step could not be off-loaded to the MLA, instead requiring floating point arithmetics and type casting on the ARM core. We replaced this quantization step with a fixed-width bitshift, which could be fused with the matrix multiplication on the MLA, see Fig. 1D. In this way, we sequentially obtained a full-precision (float32), quantized (int8) and optimized (int8+bitshift) model.

**Table 1:** High-Throughput Screening datasets used to train neural networks as quantitative structure-activity relationship (QSAR) models

Protein Target	PubChem Assay ID	No. of Molecules	
		Active	Inactive
Potassium Ion Channel Kir2.1	1843	172	301,320
KCNQ2 potassium channel	2258	287	302,191
Serine/Threonine Kinase 33	2689	172	319,619
Orexin1 Receptor	435008	234	217,924
M1 Muscarinic Receptor antagonists	435034	447	61,393
Cav3 T-type Calcium Channels	463087	703	100,171
Tyrosyl-DNA Phosphodiesterase	485290	292	341,083
Choline Transporter	488997	256	302,053
M1 Muscarinic Receptor agonists	1798	188	61,645

Molecules were curated from publicly-available PubChem assays and classified as active or inactive^60,61^.

**Fig. 2:**
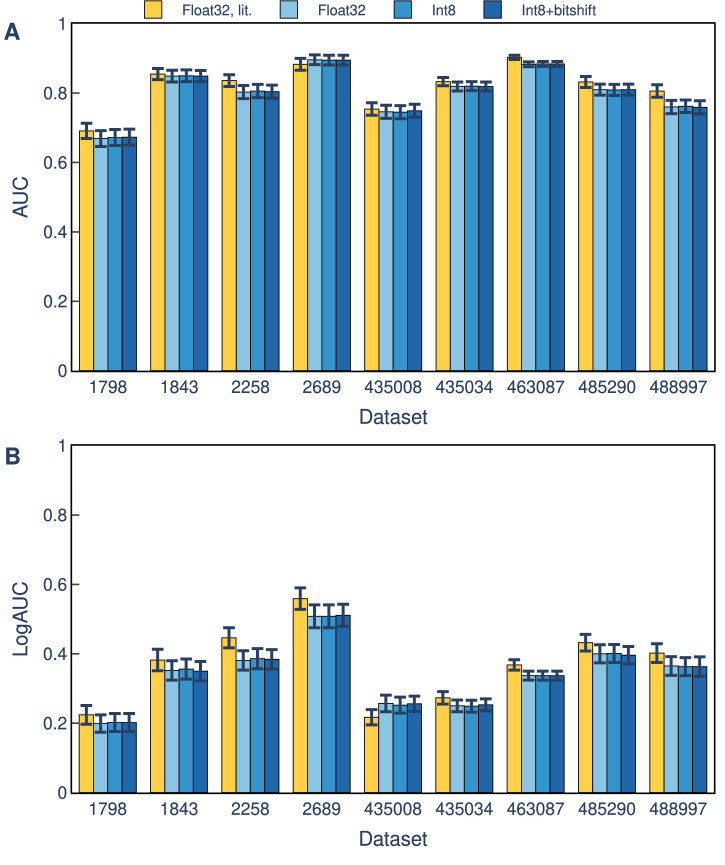
Evaluation of ANN classifiers in five-fold cross validation. **A** Area Under Curve (AUC) and **B** semi-logarithmic AUC (logAUC) of the receiver operator curve. Models in the literature (Float32, lit.) were trained in float32 precision in the Biochemical Library (BCL)^73^. We trained our models in float32 precision, then quantized them for GPU (int8) and modified them for SpiNNaker2 (int8+bitshift). Mean metrics and standard deviations were computed by bootstrapping the pooled predictions of one cross-validation 2000 times.

To evaluate the performance of our models before and after quantization, we applied the same 5-fold cross validation scheme as previous work using the same datasets^62^. Based on the pooled predictions on the 5 test sets, we calculated the area under curve (AUC) of the receiver-operator curve (ROC), as well as the semi-logarithmic AUC (logAUC)^64^, a common measure of early enrichment in drug discovery. As shown in Fig. 2, both AUC and logAUC values are in line with values reported for comparable ANNs trained on the same datasets using the BCL. Quantization and optimization for SpiNNaker2 did not significantly change AUC or logAUC on any dataset, indicating that the lowered precision after quantization did not impact predictive performance.

**Fig. 3:**
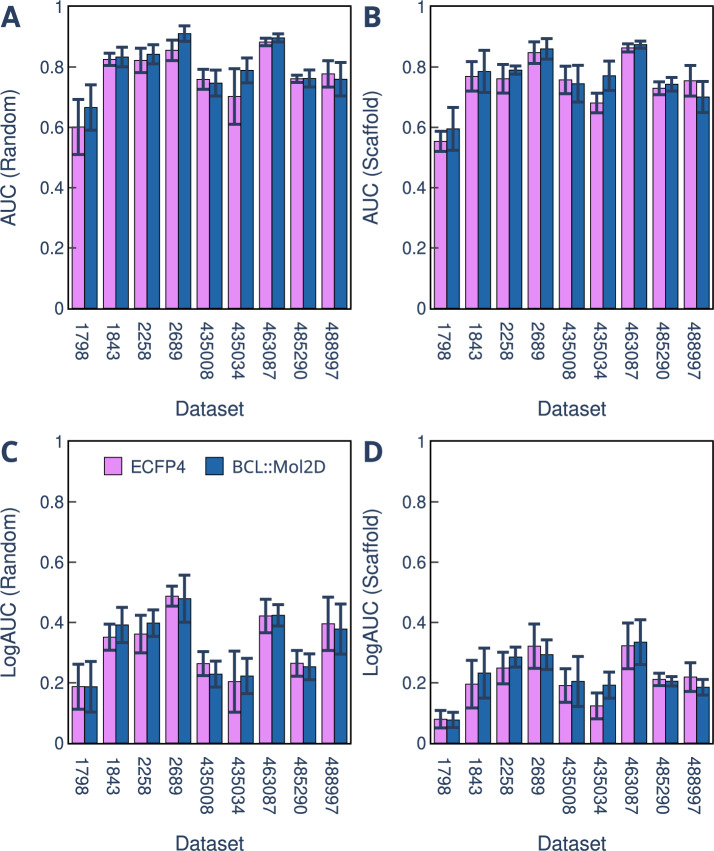
Evaluation of ANN classifiers on the WelQRate dataset in randomized and scaffold-based data splits. We used BCL::Mol2D descriptors to train and quantize our ANNs optimized for SpiNNaker2 (int8+bitshift). Additionally, we used ECFP4 descriptors to train larger ANNs in float32 precision, similar to those used as active learning components in ANN-accelerated docking campaigns. For each dataset and data split setting (random or scaffold), we report mean and standard deviation of AUC and logAUC across the five WelQRate data splits. We evaluated the area under the curve of the receiver operator curve (AUC) in **A** random split and **B** scaffold-based split setting, as well as the semi-logarithmic AUC (logAUC) in **C** random split and **D** scaffold-based split setting.

As the performance of ML-based methods is often inflated due to similarity between training and test set entries, we evaluated our quantized models on the WelQRate dataset, an updated version of the datasets above^65^. Each WelQRate dataset was released with 5 pre-defined data splits for cross-validation in a “random” setting, similar to the random data splits we applied above, and a “scaffold”-based setting. The “scaffold”-based data splits used Bemis-Murcko scaffolds to reduce molecule similarity and subsequent data leakage between training, test and validation data. Additionally, we trained larger ANNs using extended-connectivity fingerprints (ECFP4)^66,67^ as 1024-bit input vectors similar to the models previously used as active learning components in ANN-accelerated molecular docking campaigns^9,10^. For each WelQRate dataset and model type, we calculated the average AUC and logAUC across the 5 pre-defined data splits in the “random” and “scaffold” setting, as shown in Fig. 3. Model performance on the “random” data splits was largely in line with our previous cross-validation results in Fig. 2. As expected, model performance was consistently lower in the more challenging “scaffold” data split. However, the performance of our models did not differ significantly from the larger ANNs, indicating that generalizability to new molecular scaffolds remains a challenge for both our models and those used in ANN-accelerated screening campaigns.

### Descriptor calculation

**Fig. 4:**
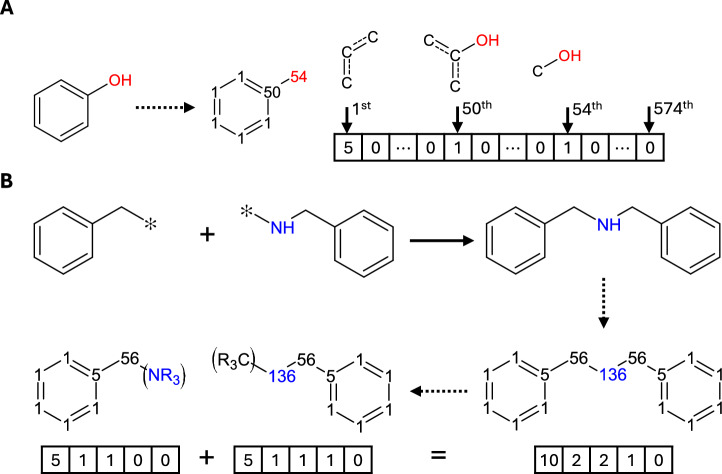
The BCL::Mol2D descriptor and our combinatorial calculation scheme. **A** Calculation of the BCL::Mol2D (Atom-1) descriptor vector of phenol as an example molecule. The identity and electronic state of each atom and its immediate neighbors are assigned as an atomic environment (AE). The descriptor vector contains the counts of 574 common AEs^62^. **B** Synthons represent molecule fragments, which can be combined through pre-defined reactions to form product molecules. AEs cannot be fully assigned to a synthon in isolation, as a new neighbor atom is introduced during the reaction. If the introduced neighbor atoms are uniform for all combinations of synthons, AEs can be fully assigned to calculate a synthon descriptor vector. Synthon vectors can be added up to compute the descriptor vector of a product molecule.

The version of the Enamine REAL space we use contains a total of 21.7 billion product molecules constructed by combining 2.22 million synthons. A simple approach to screen the combinatorial library would be to enumerate all product molecules and calculate their descriptor vectors. The calculation of a BCL::Mol2D descriptor vector for an example molecule is shown in Fig. 4A. The identity and electronic state of each atom and its immediate neighbors is treated as an atomic environment (AE) and compared to a list of 574 AEs common to drug-like molecules. The number of occurrences of each of these common AEs is stored in the descriptor vector of 574 int8 values. Pre-computing the descriptor vectors for all of these product molecules on a host system for evaluation on SpiNNaker2 would require extensive host-to-DRAM data transfer, complicating memory management and bottlenecking throughput, as shown in previous projects^56,68^.

In order to reduce the amount of data transfer needed, we introduced a descriptor calculation scheme to represent the library as descriptor vectors of synthons instead of enumerated products. Synthons represent hypothetical molecule fragments, which are combined into product molecules. Since BCL::Mol2D descriptors contain counts of AEs and each synthon atom is present in the product molecule, the descriptor vector can be viewed as the sum of AE counts from each synthon. However, AEs cannot be fully assigned to a synthon in isolation, as the combination with other synthons introduces new atoms at the reaction site. If the AEs in a synthon are the same for all synthon combinations within a reaction, they can be stored in synthon descriptor vectors, which can be added up to the descriptor vector of the corresponding product molecule. In Fig. 4B, this is shown for a benzyl and a benzylamino synthon forming a secondary amine. The AE of the aliphatic carbon in the benzyl synthon can only be assigned if the second synthon always introduces the same neighbor atom, i.e. always an sp^3^-hybridized N atom. As a test case, we applied this descriptor calculation scheme to a three-component reaction from the Enamine REAL space with 14 + 896 + 67 synthons. Instead of enumerating descriptor vectors for the resulting 840,448 product molecules, we stored descriptor vectors for the 977 synthons, reducing memory consumption from roughly 460 MB to 548 kB. For some reactions, it would be necessary to split or filter synthon lists, for example if a list contains synthons representing aliphatic amines (sp^3^-N) and alcohols (sp^3^-O). With this approach, we pre-processed the entire Enamine REAL space to obtain a library of 19 billion product molecules. As some synthons are duplicated across reactions, we obtained a total of 21.8 million synthons for a total memory consumption of 1.6 GB. Our virtual screening task is to iterate through all synthon combinations within each library reaction, sum up the corresponding synthon vectors to calculate descriptor vectors and evaluate them with one of our optimized ANNs. While this scheme is specific to BCL::Mol2D descriptors, we use it to add a CPU-bound step to our benchmark and to show the potential of distributing combinatorial libraries for screening on SpiNNaker2.

### Implementation on a SpiNNaker2 chip

Execution on the SpiNNaker2 chip is coordinated by a host system, where cross-compiled binaries for SpiNNaker2, ANN weights and input data are stored. These data are transferred through an Ethernet connection to the 2 GB DRAM (host-to-DRAM). From there, each PE can independently fetch data into its 128 kB SRAM (DRAM-to-PE). Any task running on SpiNNaker2 has to be distributed into smaller tiles per PE, which can be stored in one 128 kB SRAM for evaluation. For our virtual screening task, this included binaries, model weights and three lists of synthon vectors as well as calculated product vectors and intermediate matrix multiplication results. Quantization from float32 to int8 reduced the theoretical memory consumption of the model weights from 73.7 to 18.4 kB. In the remaining SRAM, we stored a total of 1 + 1 + 67 synthon vectors and the corresponding intermediate results to evaluate 67 product molecules at a time. Table 2 shows the actual memory footprint for this strategy on one PE with a total memory usage of 93%. Due to the small size of our ANNs and input data, more complicated memory management across PEs and higher hierarchies was not necessary. Instead, each PE was initialized with a copy of the binary and model weights and could then independently fetch synthon vectors for evaluation, until all product molecules were evaluated.

**Table 2:** Memory distribution within one processing element (PE)

Memory Block	Size [kB]	Usage [%]
synthons	45.96	35.9
weights/biases	20.50	16.0
activity	47.81	37.4
instruction code	4.77	3.7
sum	119.04	93.0

Stack and other configuration memory are omitted.

To benchmark our implementation on a SpiNNaker2 chip, we measured the CPU times for the screening of an example reaction with 840k product molecules in Table 3 with a simple and an optimized implementation on one PE, before scaling to all 152 PEs. The SpiNNaker2 offers both a low-power (150 MHz) and high-performance mode (300 MHz). We used the high-performance mode. Each implementation consisted of batchwise input preparation and inference. Inference consisted of matrix multiplication, which was off-loaded to the MLA, and a quantization step on the ARM core. We normalized all times by batch size to obtain the time spent per evaluated molecule on one PE. In our first implementation (pre-calculated descriptors), descriptor vectors were fetched from DRAM in batches of 32 for inference with our quantized model (int8) on a single PE. Fetching data from DRAM-to-PE as input preparation dominated the total runtime at 144.0 μs per evaluated molecule. Inference took 7.2 μs per evaluated molecule, the majority of which was spent on quantization steps on the ARM core. For our optimized implementation (descriptor calculation & bitshift), we introduced our scheme to calculate descriptor vectors in batches of 67 on the PE. Instead of fetching 1 + 1 + 67 synthon vectors for each batch, we kept a fixed set of 67 synthons in each PE’s SRAM and only replaced synthons in the other two positions. For inference, we used our model optimized for SpiNNaker2 (int8+bitshift), which replaced the costly int32-to-int8 quantization step between layers on the ARM core with a bitshift on the MLA. While descriptor calculation introduced an additional step on the ARM core, it reduced the amount of DRAM-to-PE data transfer and lowered the average time spent on input preparation to 9.2 μs per molecule. The optimization of our model with a hardware-accelerated bitshift reduced inference time to 1.7 μs per molecule. Compared to the initial implementation ported from traditional hardware, a more efficient library representation to reduce data transfer and an optimized ANN architecture to leverage the SpiNNaker2 hardware accelerator greatly improved total runtime.

**Table 3:** CPU times per evaluated molecule on one SpiNNaker2 processing element (PE)

Program Step		Pre-Calculated Descriptors on 1 PE		Desc. Calc. & Bitshift on 1 PE		Desc. Calc. & Bitshift on 152 PEs	
		[μs]	%	[μs]	%	[μs]	%
Input Preparation	Input fetch (DRAM-to-PE)	144.0	95.3	3.8	34.4	1.4	16.1
	Desc. calculation (ARM)	-	-	5.4	49.6	4.9	58.6
Inference	Matrix mult. (MLA)	1.3	0.8	1.5	13.9	1.5	17.4
	Quantization (ARM)	5.9	3.9	0.2	2.1	0.2	1.8
Sync	Synchronization (PE-to-PE)	-	-	-	-	0.5	6.1
Total		151.1	100	11.0	100	8.5	100

First, input was transferred from DRAM to one PE as pre-calculated descriptor vectors for evaluation with a quantized neural network in batches of 32. Next, we introduced descriptor calculation in batches of 67 to reduce memory transfer and optimized our neural network by replacing a quantization step with a bitshift operation. Finally, we scaled this implementation from one to all 152 PEs and optimized DRAM access by switching to a different DRAM interface module to mitigate additional input transfer overhead.

Finally, we scaled our optimized implementation from one PE to the entire chip, while switching DRAM interface modules for faster memory access. With all 152 PEs running in parallel, we collected CPU times while evaluating our benchmark reaction in batches of 67. Compared to the implementation on one PE, we observed an additional overhead in DRAM access, as all PEs try to access the shared DRAM interface. This was offset by the faster DRAM module, bringing time spent on input preparation to 6.3 μs. Inference remained similar to the single-PE case at 1.7 μs. Despite a small overhead introduced by PE synchronization, the more efficient memory access of our final implementation slightly reduced total screening time per molecule. During this time, the entire chip could process 152 molecules in parallel, vastly increasing throughput compared to the single-PE implementation. We used this implementation on all 152 PEs for the following benchmarks and comparisons with the Jetson Orin Nano.

### Runtime comparison

**Fig. 5:**
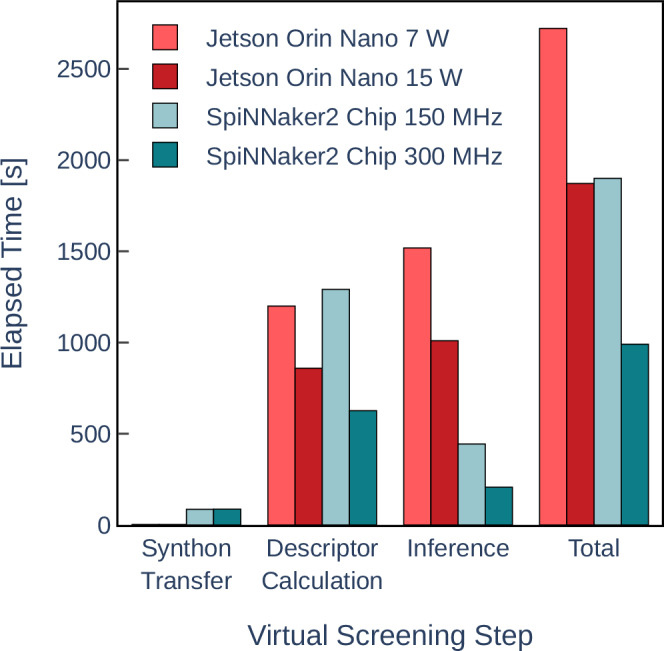
Time spent on the operations in our virtual screening of 19 billion molecules from the Enamine REAL space. Synthon Transfer is the data transfer between LPDDR4 DRAM to Processing Elements on SpiNNaker2 and SSD to RAM on the Jetson Orin Nano.

In order to compare throughput with a system of comparable size, we benchmarked the screening of our 19 billion molecule library on the SpiNNaker2 chip and a Jetson Orin Nano. We implemented descriptor calculation single-core on the ARM CPU, while inference was off-loaded to the integrated GPU. We benchmarked low-power and high-performance modes for both SpiNNaker2 chip (150, 300 MHz) and Jetson Orin Nano (7, 15 W). To compare the attainable throughput, we used the high-performance modes of both systems (300 MHz and 15 W respectively). As shown in Fig. 5, total screening time on the SpiNNaker2 chip was considerably faster at 990 s compared to 1871 s on the Jetson Orin Nano. DRAM-to-PE input fetch caused a small overhead of 86 s on the SpiNNaker2 chip. The comparable operations on the Jetson Orin Nano would be to load synthon vectors from SSD to system RAM, which took a negligible amount of time. Descriptor calculation on an ARM core took a similar amount of time on both devices at 627 s and 859 s, respectively. Inference was more than four times as fast on the SpiNNaker2 PEs at 207 s compared to 1010 s, leading to considerably higher screening throughput than on the Jetson Orin Nano.

Next to the virtual screening itself, the total runtime of a screening campaign includes steps to setup the inference and to transfer data to the SpiNNaker2 chip. In order to quantify their effect on the real use case of a full library screening, we measured the total wall clock times of running our benchmark on the Jetson Orin Nano and on the host system controlling the SpiNNaker2 chip in Table 4. As expected, the wall clock times measured for the virtual screening were approximately the same as the total screening times in Fig. 5. Only the synthon host-to-DRAM transfer to the SpiNNaker2 chip should scale linearly with the number of synthons in the library. In our benchmark, this transfer introduced an overhead of 105.7 s. Depending on the library composition and experimental setup, it might be necessary to mitigate this transfer step to take advantage of the SpiNNaker2 chip’s higher throughput, i.e. by setting up a faster host-to-DRAM Ethernet connection or keeping the same library in the DRAM for multiple screening campaigns.

**Table 4:** Wall clock times for the virtual screening of 19 billion molecules

Program step	Scales with library	SpiNNaker2		Jetson Orin Nano	
		150 MHz	300 MHz	7 W	15 W
		[s]	[s]	[s]	[s]
Runtime Setup	no	-	-	0.79	0.44
Weight Host-to-DRAM	no	0.12	0.12	-	-
Synthon Host-to-DRAM	yes	105.71	105.71	-	-
Virtual Screening	yes	1954.75	1054.02	2721.94	1871.41

Prior to the virtual screening itself, data has to be written from a host machine into the DRAM for the SpiNNaker2 chip, while the ONNX Runtime has to be initialized on the Jetson Orin Nano. Only the times for host-to-DRAM synthon transfer and screening should increase with library size.

### Power and energy consumption

**Fig. 6:**
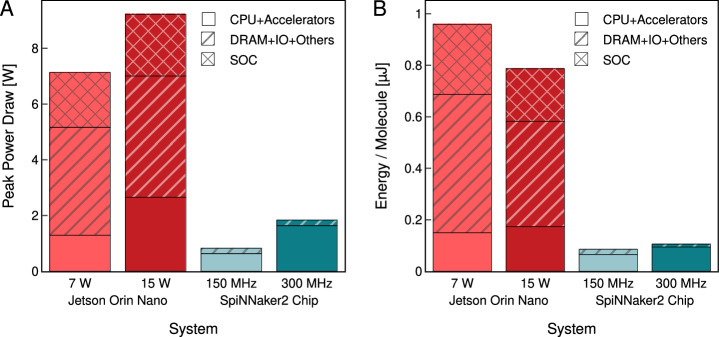
Power consumption and energy efficiency of our virtual screening on a SpiNNaker2 chip and a Jetson Orin Nano. We measured **A** peak power consumption and **B** energy consumption per evaluated product while screening a library of 19 billion molecules from the Enamine REAL space.

While energy consumption has become a point of interest for wide-spread AI application, it remains largely unreported in CADD, despite the increasing demand for computational resources. To test the potential of SpiNNaker2 to lower energy consumption in ULLS, we measured the power consumption on the SpiNNaker2 chip and the Jetson Orin Nano module using the same benchmark as above. We tested both systems in their respective low-power and high-performance modes without peripheral IO or cooling. As shown in Fig. 6A, peak power consumption was considerably lower on the SpiNNaker2 chip. The power draw of the Jetson Orin Nano stayed below its theoretical maximum, potentially because our implementation did not leverage all ARM cores and available DRAM. In either mode, the SpiNNaker2 chip power draw was at most 20% of the Jetson Orin Nano power draw. Based on the measured power draw and runtimes, we calculated the energy consumed for virtual screening by both systems, see Fig. 6B. For the total energy consumption of the SpiNNaker2 chip, we included the host-to-DRAM data transfer step, which should scale up with library size. For the Jetson Orin Nano, the higher power draw in 15 W mode was compensated by faster execution, resulting in lower energy consumption than the 7 W mode. For the SpiNNaker2 chip, the 150 MHz mode resulted in lower energy consumption, in return for lower throughput. The SpiNNaker2 Chip in 150 MHz and 300 MHz mode required 11% and 14% of the energy used by Jetson Orin Nano in 15 W mode, respectively, proving to be considerably more energy efficient.

## Discussion

Here, we present the implementation and benchmark of a ligand-based virtual library screening pipeline on a 152-core SpiNNaker2 chip. We quantized and optimized established QSAR ANNs^62^ for SpiNNaker2 and introduced a 2D combinatorial descriptor calculation to mitigate memory bandwidth bottlenecks. We benchmarked our approach by screening a set of 19 billion molecules from the Enamine REAL space on a SpiNNaker2 chip as well as a Jetson Orin Nano. As the size of ultra-large combinatorial libraries and the computational cost to screen them expand rapidly, we hypothesized that AI-based approaches tailored to the SpiNNaker2 platform could offer a way to fast and energy-efficient screening of billions of molecules.

We chose the screening of one Enamine reaction as a simple drug discovery task with a CPU-bound descriptor calculation and hardware-accelerated inference with feedforward ANNs. For the descriptor calculation, we pre-processed the synthons of the combinatorial Enamine REAL space to shift processing to the chip and reduce memory transfer. In comparison to enumerated libraries, we found that by distributing synthons among processing elements, the screening of combinatorial chemical libraries could benefit from the massively-parallel architecture of SpiNNaker2. For the inference, each SpiNNaker2 PE could process batches of molecules independently due to the small size of the ANN and 2D descriptors. Using the WelQRate dataset stratified by molecular scaffolds, we found that the predictive performance of our quantized ANN classifiers did not significantly differ from similar models used as active learning components in molecular docking campaigns^9^. As such, our implementation on SpiNNaker2 could be used for ligand-based screenings or as active learning components in docking campaigns. In the future, structure-based drug discovery algorithms on SpiNNaker2 would be of interest to fully leverage the hardware. Our optimized ANNs can serve as building blocks for the implementation of such tasks.

Any task running on SpiNNaker2 needs to be reimplemented and optimized to leverage the unique hardware architecture. We observed memory transfer to be crucial for the optimization of throughput on the SpiNNaker2 chip, most notably due to the different memory layout to the Jetson Orin Nano. Before the screening itself, the input data had to be transferred host-to-DRAM. This transfer largely depends on the Ethernet connection to the host machine and could be mitigated if the same library is screened multiple times. The equivalent data on the Jetson Orin Nano could be loaded from non-volatile storage. During the screening, DRAM-to-PE data transfer supplied the PEs with input data, similar to the transfer from system RAM to GPU memory on traditional systems. Especially for small fully-connected ANNs, this transfer has been found to bottleneck throughput^69^. A similar bottleneck was observed in previous applications of the SpiNNaker2 chip^56,68^. By using an efficient library representation and optimizing the DRAM access patterns, we could avoid this bottleneck and leverage faster inference on the SpiNNaker2 for higher throughput compared to the Jetson Orin Nano. For larger ML models with increased DRAM-to-PE and PE-to-PE transfer, “memory-only” PEs could be employed to pre-fetch and distribute data. Furthermore, we employed only one of the two DRAM interfaces, strategic use of both could double the throughput. From our implementation, we deduce specific constraints for efficient applications on SpiNNaker2: 1) External data movement, including DRAM access, should be minimized, i.e., by reusing data loaded into SRAM, 2) as many atomic operations as possible should be accelerated by the specialized hardware components, 3) execution and input needs to be tiled to fit into SRAM and ordered to maximize utilization. In this work, implementation and optimization was done manually by adapting tiling and dataflow to reduce external DRAM use, exploiting parallelization and modifying the model to use hardware-accelerated quantization and activation. A framework for multi-layer DNNs called OctopuScheduler was recently released, which automatically transforms and optimizes PyTorch models for execution on a SpiNNaker2 chip^68^. Similar domain-specific tools adapting models with minimal user input should facilitate the implementation of new models on SpiNNaker2, especially for users without extensive knowledge of the underlying system^70^.

Finally, we found that the SpiNNaker2 chip was considerably more energy efficient in executing our library benchmark. The majority of the Jetson system’s power draw and energy consumption were caused by components like the larger DRAM and non-volatile memory, which were not required for our use case, but might be relevant for other applications. However, even when comparing only the processing components of the 22nm SpiNNaker2 architecture (CPU, SRAM, accelerators) and 8nm Jetson Orin Nano (CPU, GPU), the SpiNNaker2 chip was considerably more energy efficient, an advantage that could be furthered by future ASICs using updated technology nodes. As the highly-parallel PE clusters of SpiNNaker2 were designed with energy efficiency in mind^71^, we expected to leverage this advantage through our application-specific hardware-software co-design. While power and energy consumption are usually not reported in computer-aided drug discovery projects, energy efficiency is an increasingly relevant topic in AI research, especially with the widespread adoption of generative AI. Especially large-scale industrial applications could benefit from in silico drug discovery methods designed to minimize the energy footprint and the associated cost. Our results suggest that SpiNNaker2 can present this advantage in a realistic drug discovery application. Moreover, the SpiNNaker2 chip was modeled with scalability as one of its core design principles, which should be exploited for a sizable increase in screened chemical space. With six energy efficient chip-to-chip (c2c) links and dedicated router per chip, it is possible to efficiently interlink multiple SpiNNaker2 chips in a torus-shaped network at system level, reducing node hops^49^. Already implemented boards include 48 SpiNNaker2 chips interlinked with each other. With board-to-board links (b2b) we are capable of setting up a supercomputer fit for even larger screening campaigns. In turn, it is possible to parallelize an even greater chemical space with lower energy cost. In the future, the Dresden University of Technology plans to provide remote access to a SpiNNaker2 high-performance computing cluster free of charge for academic use.

Overall, we present a drug discovery method on the neuromorphic SpiNNaker2 hardware and show its advantage over a traditional GPU-accelerated system. This advantage should be transferable to other algorithms, which can make use of the SpiNNaker2 accelerators, including the accelerator for matrix multiplication and convolution. For example, message-passing neural networks (MPNNs) should be suitable to map onto the SpiNNaker2 architecture and make use of its efficient inter-chip communications to accelerate a variety of cheminformatics and computational chemistry workflows. As application-specific hardware matures, future generations designed with virtual screening workflows in mind should widen the performance and energy-efficiency advantage over conventional hardware, offering a path for computational resources to keep up with the continued growth of make-on-demand libraries. We believe that this work will inform such software/hardware co-design and draw the attention of the community to the potential of non-conventional hardware in computer-aided drug discovery and other cheminformatics use cases.

## Materials and Methods

### SpiNNaker2 architecture

The SpiNNaker2 chip is a 2D mesh of 152 homogeneous PEs, synchronously interconnected with a 2D mesh Network-on-Chip (NoC) (see Fig. 1C). Each PE contains an ARM-Cortex M4F and heterogeneous algorithm execution modules specialized for event-based and non-event based machine learning models. For memory, the chip provides 128kByte SRAM on each PE for instruction code and data. General embedded programs run on each ARM M4F core. Due to 128kByte SRAM space per core, no operating system runs on the chip. Instead, each PE is programmed, started and controlled by a host computer sending NoC packets over SGMII Ethernet. Alternatively, a management ARM M4F core can be utilized to control the execution flow^68^. Furthermore, for more data intensive tasks, a 2 GB LPDDR4 DRAM per chip is allocated. Two DRAM direct memory access interfaces connect the LPDDR4 to the chip, with two different access modules each. Data can be transferred with SGMII UDP Ethernet to any unit inside the chip.

Among other accelerators, each PE has access to a 16 × 4 multiply-and-accumulate array, called MLA, for 8bit/16bit signed/unsigned execution of matrix multiplication and 2D convolution (conv2d), which we use to accelerate our ANNs^51,52^. The MLA can be used independently to the ARM core and an independent direct memory access unit to distribute and parallelize between these units. Each MLA furthermore provides a post-processing unit with Rectified Linear Unit activation and bitshift as possible scaling capability for quantization. To save energy, the MLA is clock-gated if data is fetched, and zero gates its rows and columns for incoming sparse data.

### Enamine REAL space

For our ULLS, we employ the Enamine REAL space, an ultra-large library based on pre-validated two- or three-component chemical reactions^27^. Each reaction is represented as a rule to combine two or three molecular building blocks, called “synthons”. Each synthon represents the molecular fragment, which an educt molecule contributes to a product molecule, with placeholders to indicate how to connect synthons in a reaction. Each reaction is associated with two or three independent lists of synthons. All products can be constructed by iterating through all combinations containing one synthon from each list, instead of storing them in an enumerated format. We use a version of the Enamine REAL space with over 21.7 billion product molecules from 280 reactions with 2.2 million synthons. As synthons are stored independently for each reaction, less than 144000 of these are unique synthons.

### QSAR datasets

We employed a set of established QSAR benchmark datasets to train ANN-based classifiers. These datasets were previously curated from publicly available high-throughput screening assays against nine diverse protein targets. Each dataset consists of small molecules encoded as SMILES strings with a binary active/inactive label and contains at least 100 active and 60,000 inactive molecules, see Table 1^60,61^. These datasets are available for download online in SDF format with a generated 3D conformer for each molecule^72^.

We used the BioChemical Library^73^ (BCL) v4.3.1 to generate BCL::Mol2D descriptor vectors for our training dataset (see Fig. 4)^62^. Each atom in a molecule was assigned an atomic environment (AE) consisting of the chemical identity and orbital configuration of the atom and its immediate neighbors. The occurrences of 574 AEs common to drug-like molecules were counted, resulting in a descriptor vector of 574 uint8 values.

### Neural network architecture

We used fully-connected feed-forward ANNs with one hidden layer of 32 neurons as QSAR models, as suggested in previous work on the same dataset^62^. Each ANN took a descriptor vector of 574 uint8 values as input and return a logit predicting whether the corresponding molecule is active or inactive. We replaced sigmoid activation in the hidden layer with ReLU, which can be executed after matrix multiplication on the SpiNNaker2 MLA without additional time cost.

The models were initialized and trained in float32 precision. The computationally most expensive steps in these networks were multiply-accumulate (MAC) operations, transforming activations **a**, weights **w** and bias **b** of each layer into an output **o**:

1 $${{{\bf{o}}}}_{{{\rm{fp32}}}}={{{\bf{W}}}}_{{{\rm{fp32}}}}{{{\bf{a}}}}_{{{\rm{fp32}}}}+{{{\bf{b}}}}_{{{\rm{fp32}}}}$$

While the SpiNNaker2 MLA can accelerate MAC operations, it only supports 16-bit or 8-bit fixed-point integer input. Similarly, the Ampere GPU of the Jetson Orin Nano supports lower-precision MAC for higher throughput. In order to leverage this hardware acceleration, trained models were quantized to int8 via quantization-aware training^63^. Symmetric per-tensor quantization from float32 to int8 was applied to both weights and activations. The scalar quantization scale *s*_*x*_ for each tensor **x** was determined during training by the largest absolute value encountered:

2 $${{{\bf{x}}}}_{{{\rm{int8}}}}={{\rm{clip}}}\left({{\rm{round}}}\left({{{\bf{x}}}}_{{{\rm{fp32}}}}/{s}_{{{\bf{x}}}}\right),-127,127\right)$$

3 $${s}_{x}=\frac{\max \left({{\rm{abs}}}\left({{{\bf{x}}}}_{{{\rm{fp32}}}}\right)\right)}{127}$$

The bias was quantized into the int32 range using the product of weight and activation scales. The output **o** was dequantized from int32 to float32:

4 $${{{\bf{o}}}}_{{{\rm{int32}}}}={{{\bf{W}}}}_{{{\rm{int8}}}}{{{\bf{a}}}}_{{{\rm{int8}}}}+{{{\bf{b}}}}_{{{\rm{int32}}}}$$

5 $${{{\bf{o}}}}_{{{\rm{fp32}}}}=\frac{{{{\bf{o}}}}_{{{\rm{int32}}}}}{{s}_{{{\bf{a}}}}\cdot {s}_{{{\bf{w}}}}}$$

The int8 MAC operation could be off-loaded to the accelerators on both systems. This quantized model (int8) was used for inference on the Jetson Orin Nano.

Since both layers expected int8 activations and returned int32 outputs, an int32-to-int8 quantization step was introduced between the two layers. In contrast to the MAC operation, this step would have been executed on the much slower SpiNNaker2 ARM core. In order to leverage the MLA for this step as well, we replaced the symmetric quantization with an arithmetic bitshift of fixed width *b*. This effectively replaced the quantization scale *s*_*x*_ with the next-largest power of 2:

6 $${b}_{x}=\lceil {\log }_{2}({s}_{x})\rceil$$

7 $${{{\bf{x}}}}_{{{\rm{int8,bitshift}}}}={{\rm{clip}}}\left({{{\bf{x}}}}_{{{\rm{int32}}}}\,\gg \, {b}_{x},-127,127\right)$$

This bitshift could be fused with the int8 MAC operation and ReLU activation on the SpiNNaker2 MLA. The resulting model (int8+bitshift) as depicted in Fig. 1D was used for inference on a SpiNNaker2 chip.

### Model training

Model training and evaluation followed previous work using the same datasets with BCL::Mol2D descriptors, unless otherwise noted^62^. For each dataset, models were trained in float32 precision by applying an AdamW optimizer to minimize binary cross entropy loss. We oversampled active samples to an active/inactive ratio of 1/10 and upweighted the loss for active samples with a factor of 10. After training for a fixed number of epochs, the trained models were copied and quantized to produce int8 and int8+bitshift models for quantization aware training. All three models were finetuned using a stochastic gradient descent optimizer with a momentum of 0.9 and a reduced learning rate for a fixed number of finetuning epochs.

To optimize hyperparameters, we applied a grid search to training epochs, finetuning epochs, learning rate and finetuning learning rate, see Table 5. For each combination of hyperparameters, we applied 5-fold cross validation and pooled predictions across test sets to obtain one prediction per sample. By bootstrapping the prediction/label pairs, we calculated mean and standard deviation of the AUC and the logAUC of the ROC. As commonly used in drug discovery tasks, the logAUC was defined as the AUC of a semi-logarithmic ROC plot within a set interval, here $FPR\in \left[0.001,0.1\right]$^64^.

**Table 5:** Hyperparameters covered in 5-fold cross validation

Hyperparameter	Search space
Learning rate	1 × 10^−2^, **5** × **10**^−**3**^
Learning rate finetuning	**5** × **10**^−**3**^, 1 × 10^−3^
Epochs	6,**8**,10
Epochs finetuning	1,**2**
Batch size	**1024**

For each combination, models quantized for SpiNNaker2 (see Fig. 1D) were trained on all datasets. The values in bold resulted in the highest average logAUC over all datasets.

Models were trained using PyTorch 2.6.0 in Python 3.12. The NVIDIA TensorRT Model Optimizer 0.27.0 (nvidia-modelopt) was used for quantization-aware training. The int8 models were quantized with default settings (INT8_DEFAULT_CFG), only input quantization of the first layer was disabled. For the int8+bitshift models, weight quantization was set to per-tensor (instead of per-channel) and the input quantization scale of the hidden layer was modified to emulate a fixed-width bitshift. Models in int8 precision were exported to ONNX format for inference on the Jetson Orin Nano.

### Model evaluation with WelQRate

To evaluate our models in a more challenging setting with mitigated data leakage, we used WelQRate, an updated version of the QSAR datasets used above^65^. Each WelQRate dataset was released with pre-defined 5-fold data splits in either a “random” or “scaffold”-based splitting scheme. “Scaffold”-based splits were generated using Bemis-Murcko scaffolds to reduce molecular similarity between molecules in the training, test and validation sets.

As in section “QSAR Datasets”, we calculated BCL::Mol2D descriptors for all datasets as input to train, quantize and finetune our ANNs in PyTorch. Additionally, we trained larger ANNs on the WelQRate datasets using ECFP4 descriptors. For the larger ANNs, we chose an architecture similar to the one used in Deep Docking consisting of multiple hidden layers, each followed by batch normalization and ReLU activation^9,74^.

For each dataset, we used the pre-defined 5-fold data splits into training, test and validation sets for cross validation. We applied the suggested cross validation scheme, whereby in each of the 5 data splits, models are trained on the training set and evaluated on the validation set in a hyperparameter grid search. The hyperparameters with the highest logAUC performance on the validation set were used to train a final model on the unified training and validation sets. This model was then evaluated on the test set to obtain an AUC and logAUC value. For each dataset and splitting scheme (random or scaffold), we obtained AUC and logAUC values for each of the five data splits to calculate AUC and logAUC mean and standard deviation.

ECFP4 descriptors were calculated with rdkit v2022.9.5. The hyperparameters shown in Table 6 were used for the hyperparameter grid search, otherwise training followed the procedure described in section “Model Training”.

**Table 6:** Hyperparameters covered in 5-fold cross validation on the WelQRate datasets

Hyperparameter	Search space BCL::Mol2D model	Search space ECFP4 model
Hidden neurons per layer	32	100, 1000
Number of hidden layers	1	2, 3
Learning rate	1 × 10^−2^, 5 × 10^−3^	1 × 10^−2^, 5 × 10^−3^
Batchnorm	No	Yes
Learning rate finetuning	5 × 10^−3^, 1 × 10^−3^	-
Epochs	6,8,10	6,8,10
Epochs finetuning	1,2	-
Batch size	1024	1024

We trained larger ANNs on ECFP4 descriptors as well as our models on BCL::Mol2D descriptors in float32 precision. We subsequently quantized and finetuned our models for SpiNNaker2.

### Combinatorial library screening

Instead of storing enumerated descriptor vectors, we introduced a descriptor calculation scheme to represent the descriptor vectors of product molecules as the sum of synthon vectors. As product molecules contain all atoms of the corresponding synthons, the descriptor vector can be viewed as the sum of AE occurrences in the synthons. If the AEs assigned in a synthon are consistent for all combinations among synthons in a reaction, AE occurrences can be pre-computed as synthon vectors.

As an example, we chose a three-component reaction with 14, 896 and 67 synthons per position for a total of 840,448 product molecules. For many other reactions in the Enamine REAL space, it was necessary to filter or split synthon lists, to ensure that the neighbor atoms of all synthons within a list are consistent. We assumed that two synthons are consistent with each other, if the atoms at the pre-defined reaction sites share the same atom type, hybridization state and bond orders. If synthons within a list differ in these properties, we split them into two or more subsets. For each subset, we created a new reaction by duplicating the remaining synthon lists. In this way, we split Enamine REAL space reactions by subdividing synthon lists, until our heuristic was satisfied. Finally, we filtered out reactions, which were still not suitable to our descriptor calculation. We obtained a library containing 19,221,855,031 product molecules constructed from 2,840,213 synthons across 510 reactions. Our benchmark was to iterate through the synthon combinations within each reaction, add up three synthon vectors to compute a descriptor vector and run inference with an ANN (see Fig. 1B). We used the reaction with 840k products to benchmark the SpiNNaker2 implementation, while we used the library with 19 billion products for the comparison of the SpiNNaker2 chip and the Jetson Orin Nano.

To assign atom environments, we picked a set of three example synthons. We combined each synthon with two example synthons into a product molecule, assigned atom environments in the product molecule and mapped them back to the synthon atoms (see Fig. 4B). For each synthon, we stored a synthon vector with the same AE counts as in BCL::Mol2D descriptor vectors. We used rdkit v2022.9.5 to parse Synthon SMILES and reaction SMARTS as well as the BCL v4.3.1 to assign atom types and AEs.

### SpiNNaker2 implementation

We benchmarked our SpiNNaker2 implementation with an example reaction of 840k molecules. For each different implementation shown in Table 3, we started by preparing the input data to be transferred, including transposing and dimension reordering. Due to 128bit access patterns of the MLA, we padded the dimensions of synthons and weights with zeros and wrote each synthon list into DRAM. We transferred the weights to each PE over Ethernet without copying data within the chip. PEs independently fetched their input data from the DRAM and executed their specific task. During matrix multiplication in the model execution, we set the ARM core in sleep mode with __WFE, while the MLA computed the biological activity of a batch of fingerprints. Output was written out from all PEs to the host over Ethernet.

To analyze bottlenecks, we first implemented and debugged the naive version of the quantized ANN model (int8) to be optimized on one PE with 300 MHz clock frequency (see Supplementary Algorithm 1). The rescaling from int32 to int8 between layers and from int32 to float32 in the output were executed on the ARM core. ReLU activation was provided by MLA post-processing. We repeated each measured step 100,000 times independently to get the mean values of ARM M4F SysTick time over loops and power. A batch size of 32 was used during that stage. The first column of Table 3 present results from said execution.

After evaluating the bottlenecks, we introduced descriptor calculation and skipped unnecessary DRAM reads by reusing synthon parts as much as possible. The accumulation of descriptor calculation was done by the ARM M4F core using its SIMD functionality with the __SADD8 function. We increased the batch size to 67 as it could still fit into PE SRAM memory and to match the list size of one of the example synthons. We again repeated each step 100,000 times and calculated mean values. Due to the optimized (int8+bitshift) model, we could now replace costly rescaling steps by doing the quantization with the MLA built-in bitshift post-processing, costing no additional power or time. To compare with our final 152 PE implementation, which did not load all 1 + 1 + 67 synthons in each batch, we normalized synthon fetch times by the number of actual DRAM accesses (see below). The optimization results are shown in the second column of Table 3.

**Fig. 7:**
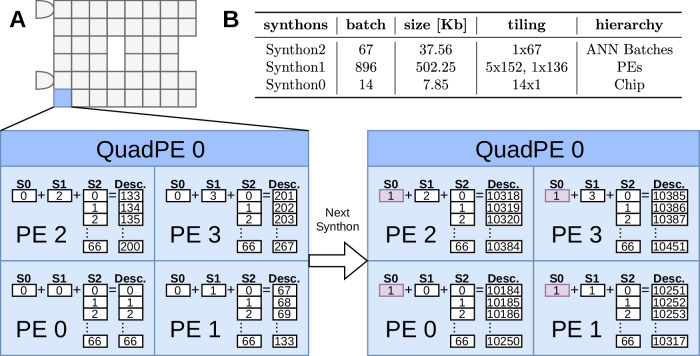
Synthon tiling strategy on SpiNNaker2. **A** Spatial representation of descriptor calculation on SpiNNaker2 for 840k combination reaction example. Each PE stored its own unique synthon tuple (S0,S1,S2) in its local SRAM and calculated 67 unique descriptors (Desc.) by summation. Each Synthon1 (S1) is unique per PE. By only replacing Synthon0 (S0) all PEs calculated 10184 new descriptor combinations. **B** Example tiling of synthon data for a 840k example reaction. Synthon lists for each reaction were reordered by size and split up by the related parallelization hierarchy. The parallelization hierarchies are intra PE (ANN Batches), inter PE and inter Chip with divisors of 67, 152 and 1 respectively. Each synthon defines 574 bytes of descriptors, zero padded up to 576 bytes to be 128bit readable.

For the third and last column of Table 3, we executed 840k combinations on all 152 PEs in parallel, looping over the full execution instead of independent steps 1000 times. This in turn slowed down DRAM access, as the access of multiple PEs now overlapped on a single resource. We mitigated this by switching to a more performant DRAM access module within the chip. Synchronization between PEs was done over a management PE, collecting a list of PE states and triggering continuation with a global feedthrough interrupt, before fetching the next synthon combination. The description of this execution is summarized in algorithm 1. As one model can be fitted to one PE, it was only necessary to consider the distribution of input data during mapping on 152 PEs. The most important part thereby was to avoid unnecessary data transfer, especially from higher memory hierarchies like the DRAM and other off-chip sources. To exploit the most out of this fact, we provided a tiling strategy adapted to the hardware (see Fig. 7), while reusing the largest synthon list as much as possible. We ordered the 2–3 synthon lists by size and mapped them to each parallelism hierarchy. We distributed the largest list (in our example synthon1) divided over 152 PEs, followed by the second-largest list (here synthon2) to the sized 67 batch dimension. Afterwards, we reordered the synthon for-loops to reduce DRAM fetch to the minimum.

To scale up to 19 billion molecules, we iterated over each reaction sequentially in their own isolated synthon-writein and inference measurement. CPU times, wall times and energy values were summed up across reactions. For this variant we neither looped over each independent step, nor over the full execution.

#### Algorithm 1

**Virtual screening of one combinatorial library reaction on a SpiNNaker2 chip with 152 PEs** 

### Benchmarking on SpiNNaker2

We benchmarked using a host PC running Ubuntu 22.04.5 LTS and compiling CPP code with gcc version 11.4.0. The ARM C code running on the SpiNNaker2 PEs was compiled with the arm-none-eabi toolchain version 10.3.1 20210621. In this work, the host PC controlled and synchronized the PEs as clients.

**Fig. 8:**
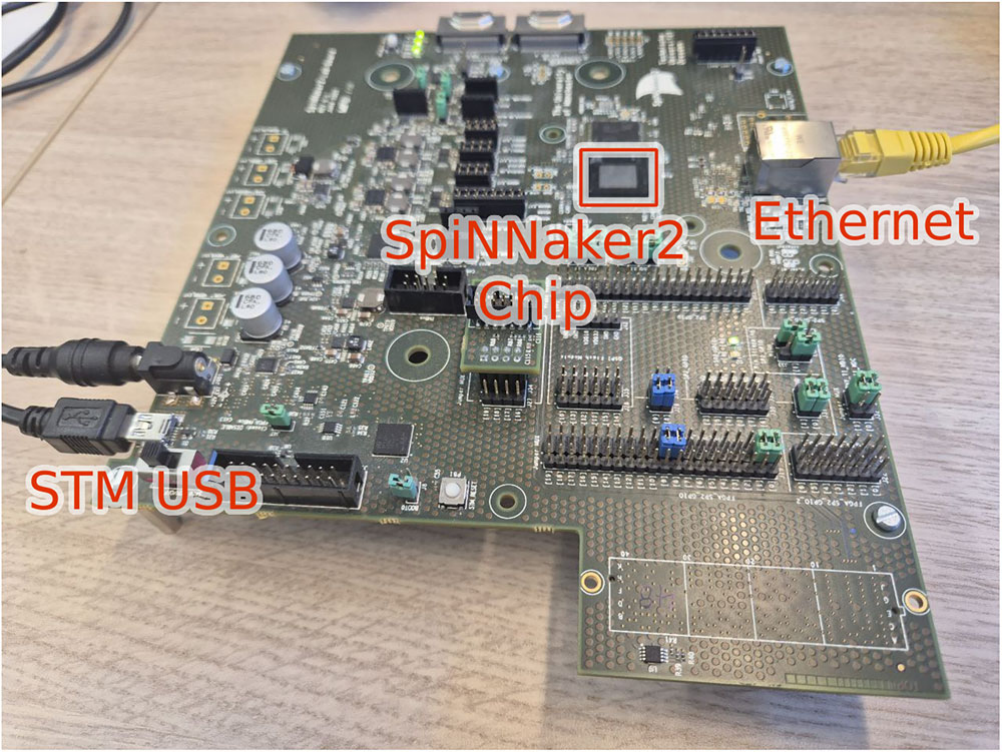
Experimental setup for the SpiNNaker2 benchmarks. The USB connects to an STM chip which reads out shunt voltages for measurements. Data is transferred over Ethernet from and to the SpiNNaker2 chip.

First, we initialized the chip and selected between two power modes, that we configured via Dynamic Voltage Frequency Scaling^75^. This is done with configuration packets we sent from a CPP program running on a host PC over Ethernet to the chip (see Fig. 8). We fixed the voltage supplies for PEs, NoC and DRAM to 0.5 V, 0.8 V and 1.1 V respectively. By configuring different clock dividers, we can supply the PEs with a frequency of either 150 MHz or 300 MHz while switching their power supply between the 0.5 V and the 0.8 V power lane, respectively. NoC and periphery frequency was fixed to 300 MHz during execution. We programmed all 152 ARM M4F cores with a C program over Ethernet, controlling execution. We set the host-PE and PE-PE communication to be event based and triggered over local interrupts or through a global feedthrough interrupt, depending on the case. Ethernet connection speed can vary depending on the hardware setup and software on the host PC. For evaluation and debugging, we relied heavily on Ethernet between each procedure steps. This is optional and not necessary during measurement and was therefore omitted.

For measuring CPU time, we utilized the ARM core M4F SysTick, while looping relevant to-be-measured steps. We chose the maximum value over PEs averaged over loops as the most realistic measurement for total time due to PEs being dependent on the last finishing PE during synchronization. For other CPU times, we present mean time. Wall time was, again, logged with clock_gettime per run with looped execution steps to get the mean. Energy recording was done by reading out shunt voltage from off-chip INA223AIDGS power monitors over time, deriving power over time (see Supplementary Fig. 1 for an 840k example reaction). The readout was done via I2C and transmitted over USB with an STM core to the host. For energy, we multiplied peak power for synthon host-to-DRAM transfer and the virtual screening process with their respective wall times.

### Benchmarking on Jetson Orin Nano

We benchmarked throughput and energy consumption on a Jetson Orin Nano 8GB developer kit running Ubuntu 20.04 and Jetpack 5.1.1 in a docker container, pulling the image dustynv/onnxruntime:1.20.2-r36.4.0 (CUDA 12.6, cuDNN 9.4.0, TensorRT 10.4.0, ONNX Runtime 1.20.2).

We implemented our virtual screening benchmark in a C program compiled with gcc 11.4.0 at optimization level 3. The ONNX Runtime was called through its C API for inference with the TensorRT execution provider. Our quantized neural networks (int8) were initialized from an ONNX model in quantize/dequantize (Q/DQ) format with int8 inference enabled and the highest TensorRT builder optimization level. Pre-processed synthon vectors were loaded from SSD into system RAM as int8 arrays representing lists of vectors. Product vectors were computed for inference in batches of a fixed size. We tested different batch sizes (512, 2048, 8192, 16,384, 32,768, 65,536, 131,072, 262,144) and found a batch size of 131,072 to result in the fastest execution.

Currents and voltages for the whole module, CPU/GPU/CV and SOC were read from sysfs nodes exposing the INA3221 power monitor every 10 ms with a C program running in parallel. We measured elapsed wall time via CLOCK_MONOTIC_RAW to obtain the time spent on different steps (ONNX runtime setup, loading synthons, descriptor calculation and inference) and to align program steps with the power measurements.

We ran our screening program once for each Jetson Orin Nano power mode to collect power draw and wall times. Each run consisted of an ONNX runtime setup (initialization at the start and memory release at the end) and the screening task (loading synthons, descriptor calculation and inference) for each reaction in the 19 billion molecule library, see Supplementary Algorithm 2. We calculated energy consumption as the product of wall time and average power draw for each reaction. Finally, we calculated total wall times and energy consumption as the sum over all reactions.

## Supplementary information

Transparent Peer Review file

Supplementary Information

Description of Additional Supplementary Files

Supplementary Data 1

## Data Availability

The HTS datasets used to train our models are available in SMILES format at https://github.com/vuoanh/BCL_Mol2D_benchmark^62^. Additionally, a newer version of the datasets with computationally generated 3D conformers in sdf format can be downloaded from https://figshare.com/articles/dataset/Well-curated_QSAR_datasets_for_diverse_protein_targets/20539893^72,76^. Access to the Enamine REAL space can be requested from Enamine Ltd. The WelQRate datasets can be accessed at http://www.welqrate.org/^65^. Numerical data for all figures can be found in Supplementary Data 1.

## Code availability

All source code for data preparation, model training, Jetson Orin Nano and SpiNNaker2 benchmarking is available under https://github.com/meilerlab/SpiNNaker2-Ultra-Large-Library-Screening. An archived version of the code can be downloaded from Zenodo via 10.5281/zenodo.20358810.
