# Supplementary material for: Rapid and energy-efficient ultra-large library screening for drug discovery on a SpiNNaker2 neuromorphic chip
Source: Commun Chem. 2026 Jul 21;9:254. doi: 10.1038/s42004-026-02122-3 (PMC13388991; doi:10.1038/s42004-026-02122-3)
Supplement: Supplementary file 2 — Supplementary Information [file 42004_2026_2122_MOESM2_ESM.pdf]

Supplementary Information for Rapid and Energy-Efficient Ultra-Large Library Screening for Drug Discovery on a SpiNNaker2 Neuromorphic Chip

Johnny Alexander Jimenez Siegert[1,2,5,*], Florian Kelber[2,7,*], Bernhard Vogginger[2,7], Paul Eisenhuth[1,2], Max Beining[1,5], Vivian Ehrlich[1], Johannes Partzsch[7], Christian Mayr[2,5,7,†,✉], and Jens Meiler[1,2,3,4,5,6,†,✉]

[1]Institute for Drug Discovery, Leipzig University, Leipzig, Germany.
[2]Center for Scalable Data Analytics and Artificial Intelligence (ScaDS.AI) Dresden/Leipzig, Germany.
[3]Center for Structural Biology, Vanderbilt University, Nashville, TN, USA.
[4]Department of Chemistry, Department of Pharmacology and Institute of Chemical Biology, Vanderbilt University, Nashville, TN, USA.
[5]School of Embedded Composite Artificial Intelligence (SECAI), Cooperation of University Leipzig and TU Dresden, Dresden/Leipzig, Germany.
[6]Faculty of Mathematics and Informatics, Faculty of Chemistry, Leipzig University, Leipzig, Germany.
[7]Institute of Circuits and Systems, Dresden University of Technology, Dresden, Germany
[*]Authors contributed equally
[†]Authors jointly supervised this work

[✉]e-mail: jens@meilerlab.org, christian.mayr@tu-dresden.de

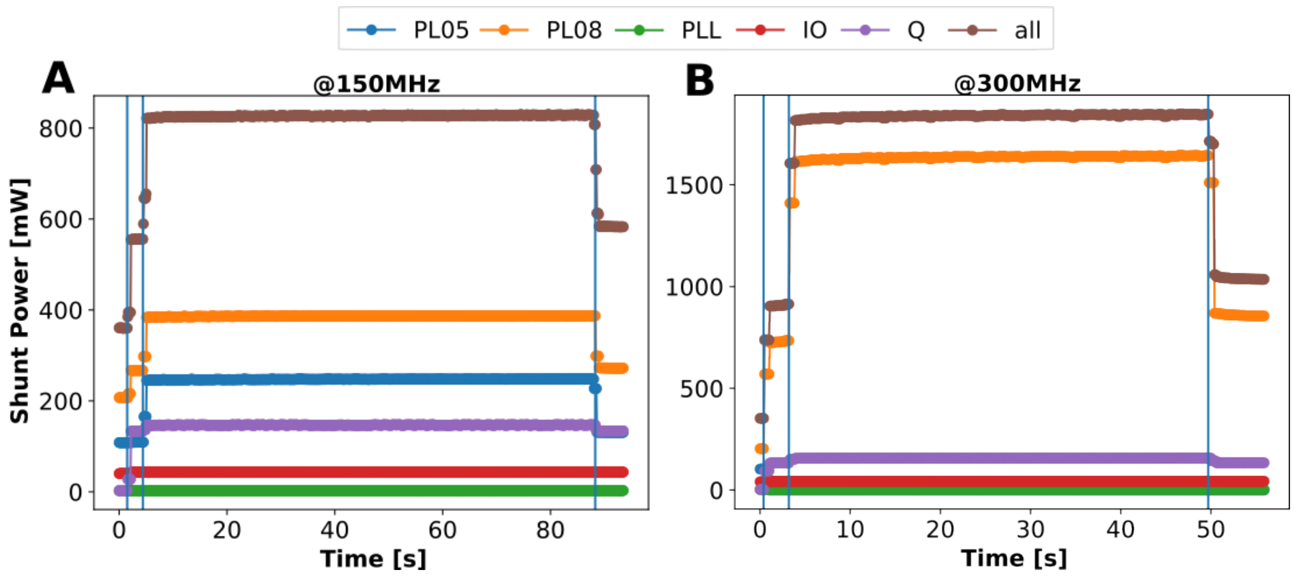

Supplementary Figure 1: Power measurement on the SpiNNaker2 for one reaction example at different power modes. Presented are **A)** 150 MHz mode and **B)** 300 MHz mode. Data was transferred host-to-DRAM at the start and the virtual screening was repeated 1000 times, while sampling power every 100 ms. PL05 and PL08 describe the power lanes 0.5 V and 0.8 V respectively. The Network on Chip is supplied by the 0.8 V power lane. For the 300 MHz case, the 0.5 V power lane is not used, as PEs are also supplied by the 0.8 V power lane. For the 150 MHz case, the PEs derive power from the 0.5 V power lane. Q, PLL and IO power are consumed for DRAM, clock generation, periphery/input/output functionality respectively.

Supplementary Algorithm 1 Virtual screening of an enumerated library on one PE of a SpiNNaker2 chip.

Require: ANN weights $\mathbf{W}$

Require: Descriptor matrix $\mathbf{D} \in \mathbb{N}^{n \times 576}$

Transfer descriptor matrix from host PC to SpiNNaker2 DRAM
Transfer weights and binaries from host PC to SpiNNaker2 PEs
Tile $\mathbf{D}$ into matrices $\mathbf{D}^a \in \mathbb{N}^{32 \times 576}$ ▷ *Evaluate descriptor vectors in batches of 32*
for all $\mathbf{D}^a$ **do**
  Fetch $\mathbf{D}^a$ from DRAM to SRAM
  Execute model $\mathbf{o} \leftarrow \text{ANN}\left(\mathbf{D}^a, \mathbf{W}\right)$
  Write out model output $\mathbf{o}$ to host PC over Ethernet

Supplementary Algorithm 2 Implementation of our virtual screening pipeline on a Jetson Orin Nano.

Require: Synthon matrices $S_0 \in \mathbb{N}^{n \times 574}$, $S_1 \in \mathbb{N}^{m \times 574}$, $S_2 \in \mathbb{N}^{l \times 574}$ for each library reaction

Require: ANN weights $\mathbf{W}$

Set up model ANN in ONNX runtime
Initialize descriptor matrix $\mathbf{D} \in \mathbb{R}^{batchsize \times 574}$
Initialize output $\mathbf{o} \in \mathbb{R}^{batchsize}$
$i \leftarrow 0$
for all Reactions (S_0, S_1, S_2) **do**
  Load synthon matrices S_0, S_1, S_2 from SSD into RAM
  ▷ *Put the smallest synthon list in the innermost loop, ideally it can be kept in cache*
  Swap S_0, S_1, S_2, such that $m > n > l$
  for all Synthon vectors $\mathbf{s_0}$ in $\mathbf{S_0}$ **do**
    for all Synthon vectors $\mathbf{s_1}$ in $\mathbf{S_1}$ **do**
      $\mathbf{p} \leftarrow \mathbf{s_0} + \mathbf{s_1}$
      for all Synthon vectors $\mathbf{s_2}$ in $\mathbf{S_2}$ **do**
        $\mathbf{D}[i] \leftarrow \mathbf{p} + \mathbf{s_2}$
        $i \leftarrow i + 1$
        if $i == batchsize$ **then**
          $\mathbf{o} \leftarrow \text{ANN}(\mathbf{D}, \mathbf{W})$
          Write $\mathbf{o}$ to disk
          $i \leftarrow 0$
  $\mathbf{o} \leftarrow \text{ANN}(\mathbf{D}, \mathbf{W})$ ▷ *Run inference on the remaining incomplete batch*
  Write $\mathbf{o}$ to disk
  Release synthon matrices S_0, S_1, S_2 from memory
Release ONNX runtime memory
