# Supplementary material for: Rapid and energy-efficient ultra-large library screening for drug discovery on a SpiNNaker2 neuromorphic chip
Source: Commun Chem. 2026 Jul 21;9:254. doi: 10.1038/s42004-026-02122-3 (PMC13388991; doi:10.1038/s42004-026-02122-3)
Supplement: Supplementary file 3 — Description of Additional Supplementary Files [file 42004_2026_2122_MOESM3_ESM.pdf]

Description of Additional Supplementary Files:

File: Supplementary Data 1

Description: Numerical data for all figures.
