# Supplementary material for: Rapid and energy-efficient ultra-large library screening for drug discovery on a SpiNNaker2 neuromorphic chip
Source: Commun Chem. 2026 Jul 21;9:254. doi: 10.1038/s42004-026-02122-3 (PMC13388991; doi:10.1038/s42004-026-02122-3)
Supplement: Supplementary file 1 — Transparent Peer Review file [file 42004_2026_2122_MOESM1_ESM.pdf]

nature portfolio

Peer Review File

Rapid and Energy-Efficient Ultra-Large Library Screening for Drug Discovery on a SpiNNaker2 Neuromorphic Chip

Corresponding Author: Mr Johnny Alexander Jimenez Siegert

This file contains all reviewer reports in order by version, followed by all author rebuttals in order by version.

Version 0:

Reviewer comments:

Reviewer #1

(Remarks to the Author)
I have not the technical competence to assess the GPU-related developments, because I am a CHEMOinformatician, and my role in this ecosystem is to make sure that the algorithms make chemical sense. If they do - then, well, results are here: the speed-up is impressive. The question, however, is exactly this: are those ANN really worth the effort of such a development? No drug discovery
y laboratory has the competence to set such a chip (tinkering with the actual board looks like open-heart surgery to application scientists...) - but it's not only that! The real problem is that targets for which you have the data needed for deep learning: really robust QSAR sets, as shown - are typically "cold" targets. Once that wealth of data becomes available, it is a sign that the target has been worked down to (a) the blockbuster on the market or (b) the understanding that it's not going to cure any disease, unfortunately. No one really cares to find muscarinic inhibitors... certainly not to the point of staging up such an impressive IT development. Also - please note that, albeit the ROC AUC values are impressive, triple caution should be applied before deeming such models to be relevant for actual drug discovery. Typically, what happens in such highly successful classification challenges is that actives all belong to one or two or three specific chemotypes, which the machine learns to distinguish - and typically predict ALL molecules with those chemotypes as "active". This is not true - but there may be significant biases in favor of actives within such "privileged" chemotypes. Say chemotype C (benzodiazepines, if you like) represents 0.1% of the database, and 10% of all molecules featuring C are active, predicting all the C as actives will mechanically produce a huge enrichment factor, even though the model has not learned any rule more sophisticated than "presence of C => active". Is this not useful? Of course it is... the catch, however, is that you do not NEED and ANN for that much. Moreover, your implementation fails to include a QSAR Applicability Domain - which is of paramount importance for the screening of ultralarge chemical spaces! GPUs are nice tools, but they do not abolish the validity of Occam's razor principle: one should use the simplest model able to reach a required performance level! And not for the sake of pure philosophy: on the contrary, real, hot drug discovery happens in a context with very sparse structure-activity data! When your only knowledge about your targets are FIVE weakly active structures, what kind of ANN would you suggest for implementation. My feeling is that you did an excellent job... all while betting on the wrong horse: before implementing ANNs on a Spinnaker chip, what about running superfast SIMILARITY searching, or SUBSTRUCTURE identification (this latter may be a real challenge!). When all you have is a hammer, everything looks like a nail! The fact that those chips are designed to run ANNs does not automatically imply that ANNs are the right approach to the real-world problem!
I believe that this contribution may, nevertheless, be published - what's done is done, and well done... but the practical concerns and limitations of this approach should be clearly stated in the Abstract, Introduction and Conclusions, and future work should be done in collaboration with real drug discoverers, out of the Ivory Tower of Pure Informatics!

Reviewer #2

(Remarks to the Author)
In this manuscript, the authors studied using the SpiNNaker2 chip to develop a neural network based on 2D molecular descriptors for virtual screening of ultra-large libraries (e.g., Enamine REAL). The authors benchmarked the performance of their hardware against the NVIDIA Jetson Orin Nano system. The results suggest that the hardware and workflow are faster and more energy efficient compared to the Jetson Orin Nano system. The manuscript provides detailed information about the performance (AUC) of the workflow on multiple protein targets and how they are compared to comparable ANNs. The efficiency benchmark results include details about the performance (wall time, memory, and power usage) of the hardware. Examples of algorithms used for virtual screening were provided.

The novelty of the work appears to be limited. For example, SpiNNaker2 and BCL::Mol2D have been introduced and discussed in earlier publications (https://arxiv.org/abs/2401.04491, 10.1007/s10822-019-00199-8). The study described in the manuscript primarily demonstrates the implementation of an existing workflow on specialized hardware, without introducing significant methodological innovation.

Additionally, the practical value of this work for the broader research community is unclear. For example, if researchers want to reproduce or try this workflow, do they need to buy this specialized hardware? Although it has been announced by SpiNNcloud Systems that the SpiNNaker2 hardware is now commercially available, it is unclear to what extent. If it is broadly available, how much does it cost? The authors should have included hardware cost in their benchmarking, as accessibility is a key consideration. For example, Anton from DESRES is a powerful hardware for running MD simulations. However, its limited availability restricts its relevance to most researchers. Similarly, the current study focuses on a specialized hardware platform that may not be widely accessible, which limits the broader impact of the findings and its relevance to scientific advancement in the field of chemistry.

Other than these main concerns, I have some minor comments:

In the introduction section, the authors make a statement “DL-based docking and co-folding methods have been suggested to replace traditional molecular docking”. I am not aware of an example of DL-based and co-folding methods that outperform traditional molecular docking in a real drug discovery project. In fact, based on my personal experience, these new methods perform much worse than traditional docking methods in studied targets. The authors need to be more careful about a statement like this because it may bias or mislead readers since these methods are a hot topic these days.

The authors mentioned that any task running on SpiNNaker2 needs to be reimplemented and optimized. If the workflow needs to be specialized every time for a new target, this is not ideal. How do the authors know the current benchmark results are representative enough to be the best performance of the workflow? The authors also failed to provide details about how to optimize the hardware for a given new target. Then as I mentioned above, this method has limited value to general users.

If the hardware is not accessible to general users, the authors need to include the cost of the hardware in their benchmark results as well. This is important to potential users who want to try the workflow.

For the reasons pointed out above, I feel that publication of the manuscript in Communications Chemistry is not suitable in its current form.

Reviewer #3

(Remarks to the Author)
I co-reviewed this manuscript with one of the reviewers who provided the listed reports. This is part of the Communications Chemistry initiative to facilitate training in peer review and to provide appropriate recognition for Early Career Researchers who co-review manuscripts.

Reviewer #4

(Remarks to the Author)
The article introduces a novel chip architecture specifically designed for ligand-based screening across ultra-large chemical spaces. The main claim of the manuscript in that this hardware solution improves efficiency compared to existing alternatives, potentially reducing the significant energy demands associated with AI-driven screening as chemical spaces continue to expand.

The work addresses an important computational bottleneck in chemical screening by proposing a hardware-based solution and the SpiNNaker2 demonstrates improved performance metrics compared to current market alternatives, which could have implications for large-scale AI screening workflows. However, there are some critical issues that should be addressed prior to publication.

Scope: The results primarily focus on hardware performance rather than chemical or methodological innovation. The appeal to a chemistry-focused readership is limited, as the discussion does not sufficiently connect the hardware improvements to chemical discovery outcomes. For example, in my opinion it is unclear how much the improvements in performance depend on the NN model used and how the use of more sophisticated would affect the scalability of the performance.

Validation: The test case involves 800K molecules, which falls within the capabilities of existing, less sophisticated methods. This raises questions about the scalability of the proposed chip.
There is insufficient analysis of accuracy and diversity in the screened molecules relative to training sets. In my view, without this, the practical impact on ligand-based screening remains unclear, and, therefore, the extension of the proposed approach to real-world research scenarios is not convincingly demonstrated.

Altogether, given the strong hardware focus and limited chemical application discussion, the manuscript would be better suited for a journal specializing in hardware development or computational architecture, rather than a chemistry-focused journal.

Version 1:

Reviewer comments:

Reviewer #1

(Remarks to the Author)
The authors have carefully discussed the points raised by the reviewers, and provided answers - in as far as there are answers, for when it comes to data sparseness there are none. This is definitely publishable - yes, but ground-breaking? no! Therefore, I suggest redirection towards a technical J. Cheminf - the perfect fit for this kind of topic.

Reviewer #2

(Remarks to the Author)
The authors have made a visible effort to address several of the concerns raised, particularly by clarifying the scope of novelty, correcting overreaching claims, and adding meaningful additional validation. Notably, the inclusion of scaffold-based evaluations and large-scale benchmarking strengthens the technical rigor of the study, and the revised framing more clearly positions the work as an exploration of hardware–software co-design rather than chemical or methodological innovation. However, despite these improvements, my core concerns regarding the practical relevance of the approach to real-world drug discovery and its suitability for a chemistry-focused readership remain only partially resolved. While the revised manuscript is clearer and more measured, its primary contribution continues to lie in specialized hardware implementation rather than advancing chemical insight or broadly applicable discovery methodology.

Reviewer #3

(Remarks to the Author)
I co-reviewed this manuscript with one of the reviewers who provided the listed reports. This is part of the Communications Chemistry initiative to facilitate training in peer review and to provide appropriate recognition for Early Career Researchers who co-review manuscripts.

Reviewer #4

(Remarks to the Author)
The authors have revised the previous version of the manuscript by increasing the test set from 800 k to 19 billion compounds. This expansion provides a much more realistic approximation of the scale encountered in contemporary ultra-large virtual screening campaigns. In my view, this strengthens the work and offers a more convincing demonstration of the chip's capabilities.
The SpiNNaker2 chip continues to show remarkable energy efficiency even under this expanded workload. Given the rapid growth of resource-intensive GPU architectures, energy consumption has become an important practical and environmental concern, and in my opinion this remains the chip's main strength. The authors also report substantial speed-ups compared to an NVIDIA chip. While this performance advantage is impressive, the ~60% increase in output should be interpreted with some caution: as screening libraries grow by orders of magnitude each year, the long-term practical impact of improvements that scale linearly rather than exponentially may be limited.
Although the manuscript has clear technical merits, I remain uncertain about its fit within the scope of Communications Chemistry. This is ultimately an editorial decision, but the hardware-focused nature of the study may sit somewhat outside the typical aims of the journal. Related to this, the authors mention plans to provide an academic-accessible cluster, which would undoubtedly broaden the impact of their technology. At the same time, they also describe ongoing efforts toward commercializing the chip. While I do not suggest any misconduct (the conflict of interest disclosure is appropriate)it is worth noting that a peer-reviewed publication may ultimately provide greater benefit to the commercial endeavor than to the academic-accessible cluster itself.
In summary, I believe the work is publishable in its current form; my only reservation is whether this particular journal is the most appropriate venue for it

Open Access This Peer Review File is licensed under a Creative Commons Attribution 4.0 International License, which permits use, sharing, adaptation, distribution and reproduction in any medium or format, as long as you give appropriate credit to the original author(s) and the source, provide a link to the Creative Commons license, and indicate if changes were made.
In cases where reviewers are anonymous, credit should be given to 'Anonymous Referee' and the source.
The images or other third party material in this Peer Review File are included in the article's Creative Commons license, unless indicated otherwise in a credit line to the material. If material is not included in the article's Creative Commons license and your intended use is not permitted by statutory regulation or exceeds the permitted use, you will need to obtain permission directly from the copyright holder.
To view a copy of this license, visit https://creativecommons.org/licenses/by/4.0/

UNIVERSITÄT LEIPZIG

Technische Universität Dresden

Johnny Alexander Jimenez Siegert, Leipzig University, Institute for Drug Discovery, Liebigstraße 21, 04103 Leipzig

Florian Kelber, Dresden University of Technology, Institute of Circuits and Systems, Mommsenstraße 12, 01069 Dresden

Leipzig, March 31, 2026

Ultra-large Library Screening on SpiNNaker2 - Manuscript Review Response

Reviewer #1 Comments

I have not the technical competence to assess the GPU-related developments, because I am a CHEMOinformatician, and my role in this ecosystem is to make sure that the algorithms make chemical sense. If they do - then, well, results are here: the speed-up is impressive. The question, however, is exactly this: are those ANN really worth the effort of such a development? No drug discovery laboratory has the competence to set such a chip (tinkering with the actual board looks like open-heart surgery to application scientists...) - but it's not only that!

Authors' response: We greatly appreciate insights from an applied cheminformatics perspective regarding both our specific implementation and the potential use of a SpiNNaker2 system in general. Ease of use is indeed a potential challenge to the adoption of SpiNNaker2. Ideally, academic users will be able to explore a SpiNNaker2 system without setting up their own chip in the future. We have added the following note to the end of the discussion (page 13, line 331):

> In the future, the Dresden University of Technology plans to provide remote access to a SpiNNaker2 high-performance computing cluster free of charge for academic use.

Additionally, future projects should benefit from the evolving SpiNNaker2 software stack to make development easier. We have modified the second paragraph of the discussion (page 12, line 300) to reflect this as follows:

> From our implementation, we deduce specific constraints for efficient applications on SpiNNaker2: 1) External data movement, including DRAM access, should be minimized i.e. by reusing data loaded into SRAM, 2) as many atomic operations as possible should be accelerated by the specialized hardware components. In this work, this was done manually by adapting the tiling and dataflow to reduce external DRAM use, exploiting parallelization and modifying the model to use hardware-accelerated quantization and activation. A framework for multi-layer DNNs called OctopuScheduler was recently released, which automatically transforms and optimizes PyTorch models for execution on a SpiNNaker2 chip.[69,71] Similar domain-specific tools adapting models with minimal user input should facilitate the implementation of new models on SpiNNaker2, especially for users without extensive knowledge of the underlying system.

The real problem is that targets for which you have the data needed for deep learning: really robust QSAR sets, as shown - are typically "cold" targets. Once that wealth of data becomes available, it is a sign that the target has been worked down to (a) the blockbuster on the market or (b) the understanding that it's not going to cure any disease, unfortunately. No one really cares to find muscarinic inhibitors... certainly not to the point of staging up such an impressive IT development.

Authors' response: We agree that the reliance of ligand-based CADD methods on experimental ligand binding data is a noteworthy aspect, usually limiting the applicability of LB-CADD methods to well-researched targets. To better explain this limitation and our motivation for choosing QSAR ANNs, we have added the following sentence to the introduction (page 2, line 52):

> Due to their reliance on target-specific binding data, LB-CADD methods are usually restricted to targets with large amounts of experimental data. QSAR models using artificial neural networks (ANNs) have recently been applied as active learning components to accelerate structure-based screening.[9–11]

Also - please note that, albeit the ROC AUC values are impressive, triple caution should be applied before deeming such models to be relevant for actual drug discovery. Typically, what happens in such highly successful classification challenges is that actives all belong to one or two or three specific chemotypes, which the machine learns to distinguish - and typically predict ALL molecules with those chemotypes as "active". This is not true - but there may be significant biases in favor of actives within such "privileged" chemotypes. Say chemotype C (benzodiazepines, if you like) represents 0.1% of the database, and 10% of all molecules featuring C are active, predicting all the C as actives will mechanically produce a huge enrichment factor, even though the model has not learned any rule more sophisticated than "presence of C =>active". Is this not useful? Of course it is... the catch, however, is that you do not NEED and ANN for that much. Moreover, your implementation fails to include a QSAR Applicability Domain - which is of paramount importance for the screening of ultralarge chemical spaces!

Authors' response: Thank you for bringing up the influence of molecular similarity on the performance and applicability of QSAR models. A similar point was raised by reviewer #4. We agree that the generalizability of QSAR models (and ML-based CADD methods in general) to structures dissimilar to their training data remains a challenge. We reported performance metrics in a randomized cross-validation setting to replicate the results previously reported with the same datasets and molecular descriptors. To address performance in a more challenging setting, we reevaluated both our models as well as larger ANNs inspired by ANN-accelerated docking campaigns on the WelQRate dataset stratified by molecular scaffolds. We have added a new figure (fig. 3) and results to subsection 2.1 (Neural Network Training and Quantization, page 4, line 138):

> As generalizability across molecular structures often remains a challenge to ML-based methods, we evaluated our quantized models on the WelQRate dataset, an updated version of the dataset above for reproducible QSAR model benchmarking. Each WelQRate dataset was released with 5 pre-defined data splits for cross-validation in a "random" and "scaffold"-based setting. The "scaffold"-based data splits used Bemis-Murcko scaffolds to reduce molecule similarity and subsequent data leakage between training, test and validation data. Additionally, we trained larger ANNs using extended-connectivity fingerprints (ECFP4)[67,68] as 1024-bit input vectors similar to the models previously used as active learning components in ANN-accelerated molecular docking campaigns.[9,10] For each WelQRate dataset and

> model type, we calculated the average AUC and logAUC across the 5 pre-defined data splits in the "random" and "scaffold" setting, as shown in fig. 3. Model performance on the "random" data splits was largely in line with our previous randomized cross-validation results in fig. 2. As expected, model performance was consistently lower in the more challenging "scaffold" data split. However, the performance of our models did not differ significantly from the larger ANNs, indicating that generalizability to new molecular scaffolds remains a challenge for both our models and those used in previous ANN-accelerated screening campaigns.

Additionally, we have added the following as subsection 4.6 (Model Evaluation with WelQRate, page 15, line 430):

> To evaluate our models in a more challenging setting with mitigated data leakage, we used WelQRate, an updated version of the QSAR datasets used above.[66] Each WelQRate dataset was released with pre-defined 5-fold data splits in either a "random" or "scaffold"-based splitting scheme. "Scaffold"-based splits were generated using Bemis-Murcko scaffolds to reduce molecular similarity between molecules in the training, test and validation sets.
>
> As above, we calculated BCL::Mol2D descriptors for all datasets as input to train, quantize and finetune our ANNs in PyTorch. Additionally, we trained larger ANNs on the WelQRate datasets using ECFP4 descriptors. For the larger ANNs, we chose an architecture similar to the one used in Deep Docking consisting of multiple hidden layers, each followed by batch normalization and ReLU activation.[9,75]
>
> For each dataset, we used the pre-defined 5-fold data splits into training, test and validation sets for cross validation. We applied the suggested cross validation scheme, whereby in each of the 5 data splits, models are trained on the training set and evaluated on the validation set in a hyperparameter grid search. The hyperparameters with the highest logAUC performance on the validation set were used to train a final model on the unified training and validation sets. This model was then evaluated on the test set to obtain an AUC and logAUC value. For each dataset and splitting scheme (random or scaffold), we obtained AUC and logAUC values for each of the 5 data splits to calculate AUC and logAUC mean and standard deviation.
>
> ECFP4 descriptors were calculated with rdkit v2022.9.5. The hyperparameters shown in table 6 were used for the hyperparameter grid search, otherwise training followed the procedure described in section 4.5.

GPUs are nice tools, but they do not abolish the validity of Occam's razor principle: one should use the simplest model able to reach a required performance level! And not for the sake of pure philosophy: on the contrary, real, hot drug discovery happens in a context with very sparse structure-activity data! When your only knowledge about your targets are FIVE weakly active structures, what kind of ANN would you suggest for implementation. My feeling is that you did an excellent job... all while betting on the wrong horse: before implementing ANNs on a Spinnaker chip, what about running superfast SIMILARITY searching, or SUBSTRUCTURE identification (this latter may be a real challenge!). When all you have is a hammer, everything looks like a nail! The fact that those chips are designed to run ANNs does not automatically imply that ANNs are the right approach to the real-world problem!

Authors' response: Thank you for raising this point. We agree that ANNs do not replace traditional LB-CADD approaches based on direct comparisons of molecular similarity or shared substructures, especially in low-data scenarios. While these ligand-based approaches would be

interesting as well, we chose ANNs as a test case with more algorithmically complex applications in mind. To contextualize our use of ANNs as QSAR models, we modified the second paragraph of the discussion (page 12, line 285) to read as follows:

> As such, our implementation on SpiNNaker2 could be used for ligand-based screenings or as active learning components in docking campaigns. In the future, structure-based drug discovery algorithms on SpiNNaker2 would be of interest to fully leverage the hardware. Our optimized ANNs can serve as building blocks for the implementation of such tasks.

I believe that this contribution may, nevertheless, be published - what's done is done, and well done... but the practical concerns and limitations of this approach should be clearly stated in the Abstract, Introduction and Conclusions, and future work should be done in collaboration with real drug discoverers, out of the Ivory Tower of Pure Informatics!

Authors' response: We are grateful for both the highlighted positive aspects and the critical feedback regarding the applicability of our method from a cheminformatics perspective. We hope that the changes we made to the manuscript and highlighted above explain both the limitations of our method and why we believe our work on SpiNNaker2 to be of interest for cheminformaticians. With the step of a first implementation taken, we hope to more closely integrate our method development on SpiNNaker2 with ongoing drug discovery campaigns.

Reviewer #2 Comments

In this manuscript, the authors studied using the SpiNNaker2 chip to develop a neural network based on 2D molecular descriptors for virtual screening of ultra-large libraries (e.g., Enamine REAL). The authors benchmarked the performance of their hardware against the NVIDIA Jetson Orin Nano system. The results suggest that the hardware and workflow are faster and more energy efficient compared to the Jetson Orin Nano system. The manuscript provides detailed information about the performance (AUC) of the workflow on multiple protein targets and how they are compared to comparable ANNs. The efficiency benchmark results include details about the performance (wall time, memory, and power usage) of the hardware. Examples of algorithms used for virtual screening were provided.

The novelty of the work appears to be limited. For example, SpiNNaker2 and BCL::Mol2D have been introduced and discussed in earlier publications (https://arxiv.org/abs/2401.04491, 10.1007/s10822-019-00199-8). The study described in the manuscript primarily demonstrates the implementation of an existing workflow on specialized hardware, without introducing significant methodological innovation.

Authors' response: We are thankful for the reviewer's comments and for raising aspects, in which we can expand and clarify our work. We acknowledge that both the SpiNNaker2 architecture as well as QSAR ANNs trained on BCL::Mol2D descriptors have been introduced before. We would argue that novelty lies in the usage of the SpiNNaker2 system, which was designed for neuromorphic computing, in a drug discovery pipeline as well as the optimizations and constraints we identified for the co-design of drug discovery methods with non-conventional hardware. To contextualize the impact of our work, we have added the following to the end of the discussion section (page 13, line 334):

> Overall, our work presents the first drug discovery method on the neuromorphic SpiNNaker2 hardware and shows its advantage over a traditional GPU-accelerated system. This advantage should be transferable to other algorithms, which can make use of the SpiNNaker2 accelerators, including the accelerator for matrix multiplication and convolution. For example, message-passing neural networks (MPNNs) should be suitable to map onto the SpiNNaker2 architecture and make use of its efficient inter-chip communications to accelerate a variety of cheminformatics and computational chemistry workflows. We believe that this work will inform future software/hardware co-design and draw the attention of the community to the potential of non-conventional hardware in computer-aided drug discovery and other cheminformatics use cases.

Additionally, the practical value of this work for the broader research community is unclear. For example, if researchers want to reproduce or try this workflow, do they need to buy this specialized hardware? Although it has been announced by SpiNNcloud Systems that the SpiNNaker2 hardware is now commercially available, it is unclear to what extent. If it is broadly available, how much does it cost? The authors should have included hardware cost in their benchmarking, as accessibility is a key consideration. For example, Anton from DESRES is a powerful hardware for running MD simulations. However, its limited availability restricts its relevance to most researchers. Similarly, the current study focuses on a specialized hardware platform that may not be widely accessible, which limits the broader impact of the findings and its relevance to scientific advancement in the field of chemistry.

Authors' response: Thank you for highlighting this concern about the access to SpiNNaker2 for researchers outside of institutions with an existing SpiNNaker2 system. Due to the different needs in hardware volume, setup and support in different cases, we cannot cite a concrete

price per SpiNNaker2 chip. As a SpiNNaker2 system can be scaled up from a single chip for development purposes to hundreds of chips in a high-performance computing cluster, the upfront cost of a system can vary greatly. However, the Dresden University of Technology intends to make development on a SpiNNaker2 system available to academic researchers free of charge, which we now indicate at the end of the discussion (page 13, line 331):

> In the future, the Dresden University of Technology plans to provide remote access to a SpiNNaker2 high-performance computing cluster free of charge for academic use.

Other than these main concerns, I have some minor comments:

In the introduction section, the authors make a statement "DL-based docking and co-folding methods have been suggested to replace traditional molecular docking". I am not aware of an example of DL-based and co-folding methods that outperform traditional molecular docking in a real drug discovery project. In fact, based on my personal experience, these new methods perform much worse than traditional docking methods in studied targets. The authors need to be more careful about a statement like this because it may bias or mislead readers since these methods are a hot topic these days.

Authors' response: Thank you for drawing our attention to this. We agree that for a variety of use cases, the usefulness of DL-based methods compared to traditional molecular docking has not been properly established. We have replaced the aforementioned point in the introduction (page 2, line 57) as follows:

> More recently, DL-based docking[16] and co-folding[17–19] methods have been suggested to predict receptor-ligand binding structures and affinities.[20] While generalizability and physical plausibility remain challenges for current generation DL methods,[21,22] their inherent treatment of protein flexibility and independence from traditional scoring functions make them a potentially useful tool in ULLS alongside molecular docking.[23,24]

The authors mentioned that any task running on SpiNNaker2 needs to be reimplemented and optimized. If the workflow needs to be specialized every time for a new target, this is not ideal. How do the authors know the current benchmark results are representative enough to be the best performance of the workflow? The authors also failed to provide details about how to optimize the hardware for a given new target. Then as I mentioned above, this method has limited value to general users.

Authors' response: Ease of use and the need to optimize applications for the SpiNNaker2 platform are indeed hurdles to its adoption. At this time, we are not aware of further optimizations that would change performance on either the SpiNNaker2 chip or the Jetson Orin Nano. As the Jetson Orin Nano implementation used the TensorRT library for inference and automatically handled memory layouts and transfers, we would expect our SpiNNaker2 implementation to have more potential for optimizations. Whether the reported performance carries over to other applications depends on the algorithm in question and will need to be benchmarked. Regarding the general approach to the implementation of future projects on SpiNNaker2, we have modified the third paragraph of the discussion (page 12, line 300) to read:

> For larger ML models with increased DRAM-to-PE and PE-to-PE transfer, "memory-only" PEs could be employed to pre-fetch and distribute data. Furthermore, we employed only one of the two DRAM interfaces, strategic use of both could double the throughput. From our implementation, we deduce specific constraints for efficient applications on SpiNNaker2: 1) External data movement, including DRAM

access, should be minimized i.e. by reusing data loaded into SRAM, 2) as many atomic operations as possible should be accelerated by the specialized hardware components, 3) execution and input needs to be tiled to fit into SRAM and ordered to maximize utilization. In this work, implementation and optimization was done manually by adapting tiling and dataflow to reduce external DRAM use, exploiting parallelization and modifying the model to use hardware-accelerated quantization and activation. A framework for multi-layer DNNs called OctopuScheduler was recently released, which automatically transforms and optimizes PyTorch models for execution on a SpiNNaker2 chip.[69,71] Similar domain-specific tools adapting models with minimal user input should facilitate the implementation of new models on SpiNNaker2, especially for users without extensive knowledge of the underlying system.

If the hardware is not accessible to general users, the authors need to include the cost of the hardware in their benchmark results as well. This is important to potential users who want to try the workflow.

Authors' response: As mentioned above, we agree that this is an important consideration for the adoption of SpiNNaker2. While we cannot cite a fixed price point for a SpiNNaker2 chip, we hope that remote access to the SpiNNaker2 machine of the Dresden University of Technology will lower the barrier of entry to method development on SpiNNaker2.

For the reasons pointed out above, I feel that publication of the manuscript in Communications Chemistry is not suitable in its current form.

Authors' response: We want to once more thank the reviewer for their time and the valuable feedback, especially regarding the relevance of our work to a chemistry-focused readership. As detailed above, we have expanded on the raised points about novelty, hardware accessibility and transferability to future projects. Ultimately, our intent is to show the considerations of hardware-aware optimization for the SpiNNaker2 platform and draw attention to the potential of non-conventional hardware for computational chemistry and cheminformatics applications. We hope that the updated version of our manuscript is clearer on these points and that you will find it suitable for a chemistry-focused readership.

Reviewer #4 Comments

The article introduces a novel chip architecture specifically designed for ligand-based screening across ultra-large chemical spaces. The main claim of the manuscript in that this hardware solution improves efficiency compared to existing alternatives, potentially reducing the significant energy demands associated with AI-driven screening as chemical spaces continue to expand.

The work addresses an important computational bottleneck in chemical screening by proposing a hardware-based solution and the SpiNNaker2 demonstrates improved performance metrics compared to current market alternatives, which could have implications for large-scale AI screening workflows. However, there are some critical issues that should be addressed prior to publication.

Scope: The results primarily focus on hardware performance rather than chemical or methodological innovation. The appeal to a chemistry-focused readership is limited, as the discussion does not sufficiently connect the hardware improvements to chemical discovery outcomes. For example, in my opinion it is unclear how much the improvements in performance depend on the NN model used and how the use of more sophisticated would affect the scalability of the performance.

Authors' response: We greatly appreciate the reviewer's feedback to our work and its appeal to a readership with a chemistry background. We acknowledge that the ANN QSAR models we used here have already been introduced previously, however we would argue that insights into the hardware-aware optimization for SpiNNaker2 and the potential of non-conventional hardware for computational drug discovery in general would be of interest to the compuational chemistry and cheminformatics community. To clarify this intention, we have modified the first paragraph of the discussion (page 12, line 278) to read as follows:

> We chose the screening of one Enamine reaction as a simple drug discovery task with a CPU-bound descriptor calculation and hardware-accelerated inference with feedforward ANNs. For the descriptor calculation, we pre-processed the synthons of the combinatorial Enamine REAL space to shift processing to the chip and reduce memory transfer. In general, distributing synthons among processing elements instead of enumerating products should allow the screening on SpiNNaker2 to leverage the combinatorial nature of chemical libraries. For the inference, each SpiNNaker2 PE could process batches of molecules independently due to the small size of the ANN and 2D descriptors. Using the WelQRate dataset stratified by molecular scaffolds, we found that the predictive performance of our quantized ANN classifiers did not significantly differ from similar models used as active learning components in molecular docking campaigns.[9] As such, our implementation on SpiNNaker2 could be used for ligand-based screenings or as active learning components in docking campaigns. In the future, structure-based drug discovery algorithms on SpiNNaker2 would be of interest to fully leverage the hardware. Our optimized ANNs can serve as building blocks for the implementation of such tasks.

Since the SpiNNaker2 performance advantage stems from the use of the machine learning hardware accelerator for matrix multiplication, we believe it to be transferable to other ML methods. While we cannot provide a detailed analysis of other neural network architectures, we have inserted a statement on constraints and optimization strategies for future methods on SpiNNaker2 in the third paragraph of the discussion (page 12, line 300):

> From our implementation, we deduce specific constraints for efficient applications on SpiNNaker2: 1) External data movement, including DRAM access, should be minimized, i.e., by reusing data loaded into SRAM, 2) as many atomic operations

> as possible should be accelerated by the specialized hardware components, 3) execution and input needs to be tiled to fit into SRAM and ordered to maximize utilization. In this work, implementation and optimization was done manually by adapting tiling and dataflow to reduce external DRAM use, exploiting parallelization and modifying the model to use hardware-accelerated quantization and activation. A framework for multi-layer DNNs called OctopuScheduler was recently released, which automatically transforms and optimizes PyTorch models for execution on a SpiNNaker2 chip.[69] Similar domain-specific tools adapting models with minimal user input should facilitate the implementation of new models on SpiNNaker2, especially for users without extensive

Additionally, we have added the following paragraph to the end of the discussion (page 13, line 334):

> Overall, our work presents the first drug discovery method on the neuromorphic SpiNNaker2 hardware and shows its advantage over a traditional GPU-accelerated system. This advantage should be transferable to other algorithms, which can make use of the SpiNNaker2 accelerators, including the accelerator for matrix multiplication and convolution. For example, message-passing neural networks (MPNNs) should be suitable to map onto the SpiNNaker2 architecture and make use of its efficient inter-chip communications to accelerate a variety of cheminformatics and computational chemistry workflows. We believe that this work will inform future software/hardware co-design and draw the attention of the community to the potential of non-conventional hardware in computer-aided drug discovery and other cheminformatics use cases.

Validation: The test case involves 800K molecules, which falls within the capabilities of existing, less sophisticated methods. This raises questions about the scalability of the proposed chip.

Authors' response: We agree that a benchmark on the scale of the full Enamine REAL space is preferable to more conclusively show performance in a realistic use case. While we believe our initial test case of 840,448 molecules to be enough to be identify significant differences in performance and energy efficiency, this relied on the assumption that our results would scale to the full library. Therefore, we have rerun our benchmarks with a considerably larger part of the Enamine REAL space covering roughly 19 billion molecules. We have updated the results presented in fig. 5, fig. 6 and table 4 as well as the numbers and percentages cited from these results throughout the result section. As these results did not change the trends in comparison between Jetson Orin Nano and SpiNNaker2 chip, our conclusions in the result and discussion sections remain unaffected.

We have modified the subsections 4.8 (SpiNNaker2 Implementation, page 16, line 473) and 4.10 (Benchmarking on Jetson Orin Nano, page 18, line 552) to reflect that we now use one run of our screening program on the 19 billion molecule library instead of repeated runs on the 840k example reaction for our benchmarking. Additionally, we have added information on our expanded library to subsection 4.2 "Enamine REAL Space" (page 15, line 454):

> As an example, we chose a three-component reaction with 14, 896 and 67 synthons per position for a total of 840 448 product molecules. For many other reactions in the Enamine REAL space, it was necessary to filter or split synthon lists, to ensure that the neighbor atoms of all synthons within a list are consistent. We assumed that two synthons are consistent with each other, if the atoms at the pre-defined reaction sites share the same atom type, hybridization state and bond orders. If synthons within a list differ in these properties, we split them into two or more subsets. For each subset, we created a new reaction by duplicating the remaining

> synthon lists. In this way, we split Enamine REAL space reactions by subdividing synthon lists, until our heuristic was satisfied. Finally, we filtered out reactions, which were still not suitable to our descriptor calculation. We obtained a library containing 19,221,855,031 product molecules constructed from 2,840,213 synthons across 510 reactions. Our benchmark is to iterate through the synthon combinations within each reaction, add up three synthon vectors to compute a descriptor vector and run inference with an ANN (see fig. 1B). We used the reaction with 840k products to benchmark the SpiNNaker2 implementation, while we used the library with 19 billion products for the comparison of the SpiNNaker2 chip and the Jetson Orin Nano.

There is insufficient analysis of accuracy and diversity in the screened molecules relative to training sets. In my view, without this, the practical impact on ligand-based screening remains unclear, and, therefore, the extension of the proposed approach to real-world research scenarios is not convincingly demonstrated.

Authors' response: Thank you for bringing our attention to the influence of molecular similarity on the performance of QSAR models (and ML-based CADD methods in general). A similar point was raised by reviewer #1. We agree that molecular similarity to training set molecules can inflate model performance, which might imply a generalizability that is not transferable to real-world applications. Initially, we performed only randomized cross-validation to show that the performance of our quantized model did not differ significantly from previous results using the same datasets and methodology. To address performance in a more realistic setting, we reevaluated our models on the WelQRate dataset stratified by molecular scaffolds and added a new figure (fig. 3) as well as the following paragraph to subsection 2.1 (Neural Network Training and Quantization):

> As generalizability across molecular structures often remains a challenge to ML-based methods, we evaluated our quantized models on the WelQRate dataset, an updated version of the dataset above for reproducible QSAR model benchmarking. Each WelQRate dataset was released with 5 pre-defined data splits for cross-validation in a "random" and "scaffold"-based setting. The "scaffold"-based data splits used Bemis-Murcko scaffolds to reduce molecule similarity and subsequent data leakage between training, test and validation data. Additionally, we trained larger ANNs using extended-connectivity fingerprints (ECFP4)[67,68] as 1024-bit input vectors similar to the models previously used as active learning components in ANN-accelerated molecular docking campaigns.[9,10] For each WelQRate dataset and model type, we calculated the average AUC and logAUC across the 5 pre-defined data splits in the "random" and "scaffold" setting, as shown in fig. 3. Model performance on the "random" data splits was largely in line with our previous randomized cross-validation results in fig. 2. As expected, model performance was consistently lower in the more challenging "scaffold" data split. However, the performance of our models did not differ significantly from the larger ANNs, indicating that generalizability to new molecular scaffolds remains a challenge for both our models and those used in previous ANN-accelerated screening campaigns.

Altogether, given the strong hardware focus and limited chemical application discussion, the manuscript would be better suited for a journal specializing in hardware development or computational architecture, rather than a chemistry-focused journal.

Authors' response: We would like to thank the reviewer for engaging with our work and for the evaluation of its content and impact. As mentioned above, we believe that the co-

design of computational chemistry and cheminformatics methods with specialized hardware are of interest to the community and that our work can give insights into the potential of this direction. We hope that the updated manuscript makes a stronger case for this view and that you will find it of interest to a chemistry-focused readership.

Table 1: Hyperparameters covered in 5-fold cross validation on the WelQRate datasets. We trained larger ANNs on ECFP4 descriptors as well as our models on BCL::Mol2D descriptors in float32 precision. We subsequently quantized and finetuned our models for SpiNNaker2.

**Hyperparameter**	**Search space** BCL::Mol2D model	**Search space** ECFP4 model
Hidden neurons per layer	32	100, 1000
Number of hidden layers	1	2, 3
Learning rate	1×10^{-2}, 5×10^{-3}	1×10^{-2}, 5×10^{-3}
Batchnorm	No	Yes
Learning rate finetuning	5×10^{-3}, 1×10^{-3}	-
Epochs	6,8,10	6,8,10
Epochs finetuning	1,2	-
Batch size	1024	1024

Table 4: Wall clock times for the virtual screening of 19 221 855 031 molecules. Prior to the virtual screening itself, data has to be written from a host machine into the DRAM for the SpiNNaker2 chip, while the ONNX Runtime has to be initialized on the Jetson Orin Nano. Only the times for host-to-DRAM synthon transfer and screening should increase with library size.

Program Step	Scales with library	SpiNNaker2		Jetson Orin Nano	
		150 MHz [s]	300 MHz [s]	7 W [s]	15 W [s]
Runtime Setup	no	-	-	0.79	0.44
Weight Host-to-DRAM	no	0.12	0.12	-	-
Synthon Host-to-DRAM	yes	105.71	105.71	-	-
Virtual Screening	yes	1954.75	1054.02	2721.94	1871.41

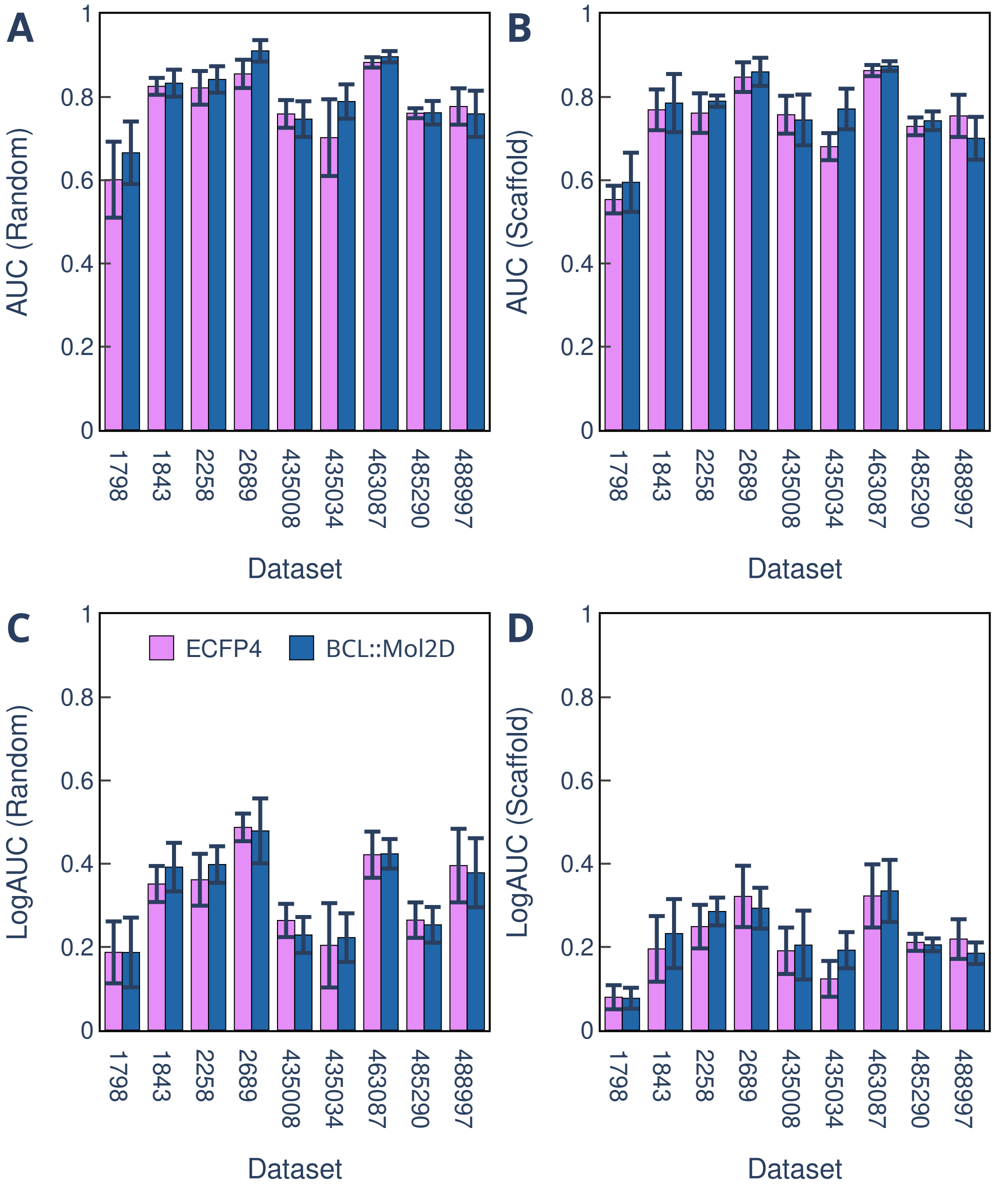

Figure 3: Evaluation of ANN classifiers on the WelQRate dataset in A,B) randomized and C,D) scaffold-based data splits. We trained our quantized ANNs on Mol2D descriptors as well as larger ANNs on ECFP4 descriptors, similar to those used as active learning components in previous docking campaigns. For each dataset and data split (random or scaffold), we report mean and standard deviation of AUC and logAUC across the five WelQRate data splits.

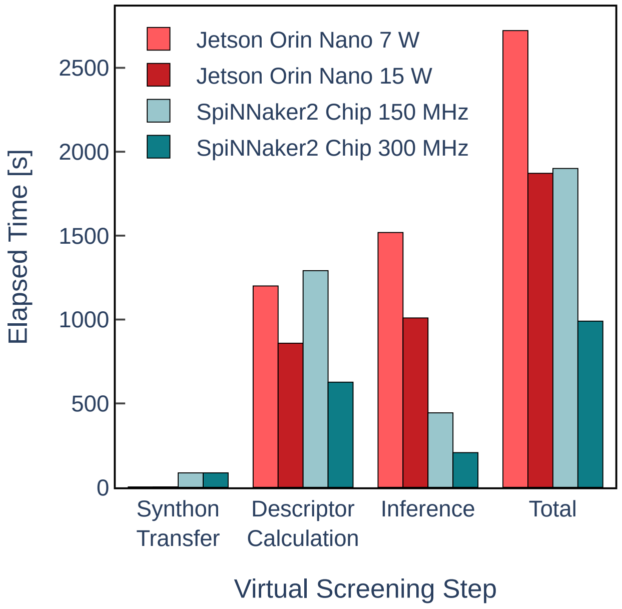

Figure 5: Time spent on the operations in our virtual screening of 19 billion molecules from the Enamine REAL space. Synthon Transfer is the data transfer between LPDDR4 DRAM to Processing Elements on SpiNNaker2 and SSD to RAM on the Jetson Orin Nano.

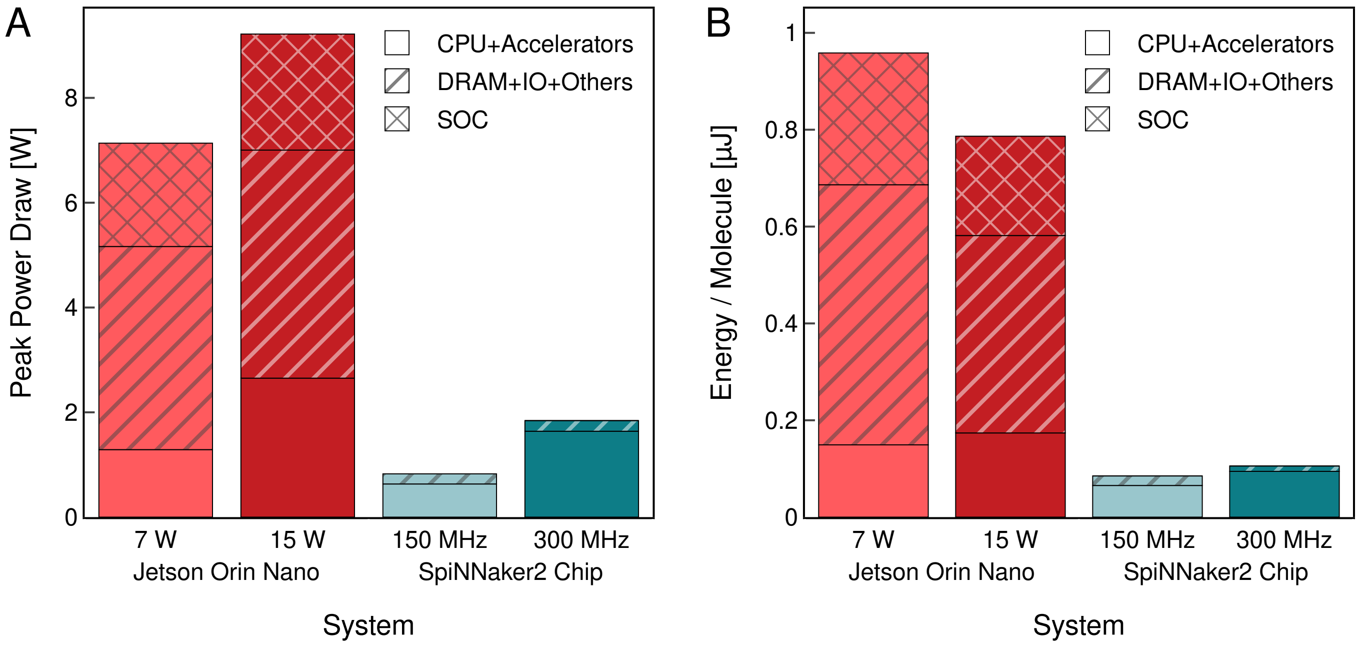

Figure 6: A) Peak power consumption and B) energy consumption per evaluated product during the virtual screening of 19 billion molecules from an Enamine REAL space reaction.

Johnny Alexander Jimenez Siegert, Leipzig University, Institute for Drug Discovery, Liebigstraße 21, 04103 Leipzig
Florian Kelber, Dresden University of Technology, Institute of Circuits and Systems, Mommsenstraße 12, 01069 Dresden

Leipzig, May 23, 2026

Ultra-large Library Screening on SpiNNaker2 - Manuscript Review Response

Reviewer #4 Comments

The authors have revised the previous version of the manuscript by increasing the test set from 800 k to 19 billion compounds. This expansion provides a much more realistic approximation of the scale encountered in contemporary ultra-large virtual screening campaigns. In my view, this strengthens the work and offers a more convincing demonstration of the chip's capabilities.

The SpiNNaker2 chip continues to show remarkable energy efficiency even under this expanded workload. Given the rapid growth of resource-intensive GPU architectures, energy consumption has become an important practical and environmental concern, and in my opinion this remains the chip's main strength. The authors also report substantial speed-ups compared to an NVIDIA chip. While this performance advantage is impressive, the 60% increase in output should be interpreted with some caution: as screening libraries grow by orders of magnitude each year, the long-term practical impact of improvements that scale linearly rather than exponentially may be limited.

Authors' response: We agree that the SpiNNaker2 architecture on its own does not offer a permanent solution to the growing demands of ultra-large library screening. While suitable models could benefit even further from the SpiNNaker2 chip's hardware features, there will be a ceiling to the architecture's advantage in throughput. However, we believe that future generations of neuromorphic hardware and specialized hardware in general might allow computational resources to keep pace with the growing computational demands of virtual screening. To clarify this, we have added the following sentence to the last paragraph of the discussion (page 7, line 337):

> As application-specific hardware matures, future generations designed with virtual screening workflows in mind should widen the performance and energy-efficiency advantage over conventional hardware, offering a path for computational resources to keep up with the continued growth of make-on-demand libraries.

Although the manuscript has clear technical merits, I remain uncertain about its fit within the scope of Communications Chemistry. This is ultimately an editorial decision, but the hardware-focused nature of the study may sit somewhat outside the typical aims of the journal. Related to this, the authors mention plans to provide an academic-accessible cluster, which would undoubtedly broaden the impact of their technology. At the same time, they also describe ongoing efforts toward commercializing the chip. While I do not suggest any misconduct (the conflict of interest disclosure is appropriate)it is worth noting that a peer-reviewed publication

may ultimately provide greater benefit to the commercial endeavor than to the academic-accessible cluster itself.

Authors' response: Thank you for raising this point. It was certainly not our intention to give undue emphasis to the commercialization of the SpiNNaker2 hardware. To prevent any misunderstanding in this direction, we have removed the mention of the development of further clusters by SpiNNCloud Systems from the discussion (page 7, line 331). We have also removed the mention of the "5-million-core SpiNNcloud system" from the abstract (page 1, line 28), which could be read as a system operated by SpiNNcloud systems, but in actuality refers to a SpiNNaker2 cluster operated by the Technical University of Dresden. We hope that these changes help clarify the manuscript and preempt any potential concerns.

In summary, I believe the work is publishable in its current form; my only reservation is whether this particular journal is the most appropriate venue for it

Authors' response: We would again like to thank the reviewer for their insights, which have helped us in clarifying the manuscript and improving our work.
